# Development and Validation of Questionnaires Exploring Health Care Professionals' Intention to Use Wiki-Based Reminders to Promote Best Practices in Trauma

**DOI:** 10.2196/resprot.3762

**Published:** 2014-10-03

**Authors:** Patrick Michel Archambault, Susie Gagnon, Marie-Pierre Gagnon, Stéphane Turcotte, Jean Lapointe, Richard Fleet, Mario Côté, Pierre Beaupré, Natalie Le Sage, Marcel Émond, France Légaré

**Affiliations:** ^1^Département de médecine familiale et médecine d'urgenceUniversité LavalQuébec, QCCanada; ^2^Centre de santé et de services sociaux Alphonse-Desjardins (Centre hospitalier affilié universitaire de Lévis)Lévis, QCCanada; ^3^Division de soins intensifsUniversité LavalQuébec, QCCanada; ^4^Centre de recherche du CHU de QuébecAxe Santé des populations - Pratiques optimales en santé, Traumatologie – Urgence – Soins IntensifsQuébec, QCCanada; ^5^Faculté des sciences infirmièresUniversité LavalQuébec, QCCanada; ^6^Institut national d'excellence en santé et services sociauxMontreal, QCCanada; ^7^Canada Research Chair in Implementation of Shared Decision Making in Primary CareUniversité LavalQuébec, QCCanada

**Keywords:** knowledge translation, wiki, collaborative writing applications, decision support tools, health informatics, Theory of Planned Behavior, trauma care, traumatic brain injury, interprofessional collaboration

## Abstract

**Background:**

Little is known about factors influencing professionals’ use of wikis.

**Objective:**

We developed and validated two questionnaires to assess health care professionals’ intention to use wiki-based reminders for the management of trauma patients.

**Methods:**

We developed questionnaires for emergency physicians (EPs) and allied health professions (AHPs) based on the Theory of Planned Behavior and adapted them to the salient beliefs of each, identified in an earlier study. Items measured demographics and direct and indirect theoretical constructs. We piloted the questionnaires with 2 focus groups (5 EPs and 5 AHPs) to identify problems of wording and length. Based on feedback, we adjusted the wording and combined certain items. A new convenience sample of 25 EPs and 26 AHPs then performed a test-retest of the questionnaires at a 2-week interval. We assessed internal consistency using Cronbach alpha coefficients and temporal stability of items with an agreement intraclass correlation coefficient (ICC).

**Results:**

Five EPs and 5 AHPs (3 nurses, 1 respiratory therapist, and 1 pharmacist) formed 2 focus groups; 25 EPs and 26 AHPs (12 nurses, 7 respiratory therapists, and 7 pharmacists) completed the test and retest. The EP questionnaire test-retest scores for consistency (Cronbach alpha) and stability (ICC) were intention (test: Cronbach alpha=.94; retest: Cronbach alpha=.98; ICC=.89), attitude (.74, .72, .70), subjective norm (.79, .78, .75), perceived behavioral control (.67, .65, .66), attitudinal beliefs (.94, .86, .60), normative beliefs (.83, .87, .79), and control beliefs barriers (.58, .67, .78) and facilitators (.97, .85, .30). The AHP questionnaire scores for consistency and stability were: intention (test Cronbach alpha=.69, retest Cronbach alpha=.81, ICC=.48), attitude (.85, .87, .83), subjective norm (.47, .82, .62), perceived behavioral control (.55, .62, .60), attitudinal beliefs (.92, .91, .82), normative beliefs (.85, .90, .74), and control beliefs barriers (.58, .55, .66) and facilitators (.72, .94, –.05). To improve the psychometric properties of both questionnaires, we reformulated poorly consistent or unstable items.

**Conclusions:**

Our new theory-based questionnaires to measure health care professionals’ intention to use wiki-based reminders have adequate validity and reliability for use in large surveys. In the long run, they can be used to develop a theory-based implementation intervention for a wiki promoting best practices in trauma care.

## Introduction

Clinical practice does not always reflect best evidence. High proportions of inappropriate care have been reported in different health care systems and settings [1]. This has a huge impact on both patient outcomes and health care costs. As passive dissemination of evidence has not proven adequate for encouraging implementation of research-based recommendations for changes in practice, new strategies are being advocated [2].

Information and communication technologies (ICTs) such as computerized decision support systems have been suggested as a possible solution for improving research uptake and increasing evidence-based practice [3]. Aiming to improve care and reduce costs, governments have invested billions of dollars to implement ICTs, including decision support systems, but these systems have yet to deliver the expected benefits [4]. Moreover, some health care professionals have rejected ICTs on the grounds that they are slow, incompatible with work processes, difficult to access, costly to implement, and cannot be adapted to local practices [4-10]. Furthermore, local initiatives to adapt various ICT solutions seem to be restricted to a small number of hospitals and tools are mostly designed for local use only [11-13]. Transfer of these local initiatives to the larger health care community is often slow and complex. In emergency departments (EDs), where shift work is prevalent, getting health care professionals to collaborate in creating, using, and updating decision support tools (eg, care protocols, care pathways, and decision aids) is particularly difficult [14]. These decision support tools can be translated into paper-based or computer-based reminders that support clinicians’ or patients’ decision making at the bedside. The most important factors influencing the creation, use, and updating of any form of reminders to promote best practices may be time and collaboration within and across care teams [15,16]. Wikis are an open-source and low-cost means of accelerating innovation and permitting a broad spectrum of stakeholders to collaborate efficiently for this purpose.

Wikis are knowledge management platforms that empower stakeholders to implement evidence-based decision support tools in different areas of health care [17]. A wiki is a website that uses a novel technology to allow people to view and edit website content, with viewing and editing privileges determined by various levels of access. Wikipedia—the best-known wiki—has 365 million visitors per month, is the sixth most popular website in the world, and its medical articles (available in 271 languages) are viewed approximately 150 million times per month [18]. Many health organizations have started using wikis to manage knowledge and coordinate care [19-23]. A recent scoping review found that wikis are effective educational interventions for health students and professionals and that they have many positive impacts on knowledge translation processes and outcomes: theoretical behavioral change domains (eg, beliefs about capabilities), learning (eg, skills and knowledge), communication, collaboration, knowledge management, health care efficiency, quality improvement, and disease prevention [17].

A wiki could permit stakeholders in 1 or many EDs to collaborate asynchronously in the updating and creation of reminders, decreasing duplication efforts and reducing the time needed. However, despite increasing evidence supporting the use of wikis in various settings, there is a lack of knowledge about the factors influencing professionals’ use of wikis and about how best to implement them in health care settings [24,25].

The objectives of this study were to develop and test the psychometric properties of 2 questionnaires based on the Theory of Planned Behavior (TPB) [26] exploring the intention of ED health care professionals and the determinants of this intention to use wiki-based reminders promoting best practices for the management of severe traumatic brain injury (TBI) victims.

##  Methods

### Study Design

The protocol for this mixed methods study describes 4 phases [27]: (1) eliciting salient beliefs [25], (2) developing the questionnaires, (3) piloting the questionnaires, and (4) test-retest of the adjusted questionnaires. Phase 1 of this project identified ED professionals’ salient beliefs concerning the use of wiki-based reminders promoting best practices for the management of severe TBI [25]. The current study represents the later phases (2, 3, and 4) of the published research protocol [27]. Our participants, emergency physicians (EPs) and allied health professionals (AHPs), came from 3 hospitals of 3 different trauma levels (I, II, and III) in the province of Quebec, Canada. All our participants were French speaking. The ethics committees from the 3 hospitals approved this study and there were no financial incentives offered to participants.

### Definition of the Behavior for the Present Survey

We chose to study the intention (and determinants of intention) of ED health care professionals to use a wiki-based reminder promoting best practices for the management of severe TBI victims in the ED in the Province of Quebec, Canada. Definition of the behavior was:

1. Action: to use
2. Target: a wiki-based reminder promoting best practice
3. Context: management of severe TBI victims in EDs in the province of Quebec, Canada

### Phase 1: Elicitation of Salient Beliefs

A complete report of Phase 1 of our study has been published [25]. In summary, we conducted semistructured interviews to elicit EPs’ and AHPs’ beliefs about using a wiki-based reminder. In order to clearly depict the behavior being studied, 4 videos were created presenting 4 different health care professionals (emergency physician, nurse, respiratory therapist, and pharmacist) performing the behavior (see Multimedia Appendix 1 to access the YouTube videos in French). After watching the video specific to their profession, each participant was interviewed about their behavioral, control, and normative beliefs (ie, what they saw as advantages, disadvantages, barriers, and facilitators to their use of a wiki-based reminder) and how they felt important referents would perceive their use of a wiki-based reminder. After ranking each belief from the most reported to the least reported, we considered the top 75% most-reported beliefs as salient. We also retained certain beliefs as salient although they were not among the top 75% most reported. This decision was based on our knowledge of the literature, our experience in implementing care protocols for trauma, or our fear of excluding important negative beliefs. This study generated 2 different sets of salient beliefs for EPs and AHPs that were used to construct 2 different questionnaires.

### Phase 2: Questionnaire Development

#### Direct Construct Items

In both questionnaires, we included items to measure the constructs identified in our theoretical model: intention (n=3), perceived behavioral control (n=3), attitude (n=4), and subjective norm (n=3). The items were formulated so that participants could then evaluate their level of agreement with each statement on a 7-point Likert scale.

#### Indirect Construct Items

We selected the salient behavioral, normative, and control beliefs identified in our published Phase I and converted these into a set of statements for each questionnaire. The items were formulated so that participants could evaluate their level of agreement on a 7-point Likert scale about each advantage, disadvantage, positive referent, negative referent, barrier, and facilitator presented.

#### Characteristics of Health Care Professionals

We assessed the following demographic characteristics: age, gender, type of health care professional, and diploma (AHP questionnaire), training level of EPs (EP questionnaire), type of health care center (level I, II, III), number of years in practice, presence of computers with unrestricted access to the Internet within their ED, availability of Wi-Fi for professionals, availability of Wi-Fi for patients, previous consultation of or contribution to a wiki, and membership in local trauma committees.

#### Ordering of Questions in the Questionnaires

The drafts of our initial questionnaires were created without randomly mixing the items and in the following order: intention, perceived behavioral control, subjective norm, attitude, control beliefs (facilitators), control beliefs (barriers), normative beliefs, and attitudinal beliefs (advantages and disadvantages).

### Phase 3: Pilot Testing of the Questionnaires

We pilot tested our questionnaires by asking a convenience sample of 10 participants (5 physicians and 5 AHPs) from our population to answer the questionnaire intended for their own professional group before the focus group. Participants for this phase were recruited purposefully to represent a wide range of professionals and to represent different age groups. Participants could choose either a paper-based survey or a Web-based survey (SurveyMonkey). This choice was meant to ensure that participants less comfortable with computers could access a paper-based version of our questionnaire. We formed 2 focus groups (EPs in 1 and AHPs in the other) to tell us whether they had any difficulty answering the questions. The Web-based survey contained an HTML link to a YouTube video presenting the behavior being studied and paper-based participants were sent the link in the invitation email. For participants who had not completed the survey before the focus group, we presented the video during the focus groups and then they answered a paper-based questionnaire.

We then interviewed participants about both the Web-based and paper-based questionnaires to check comprehension and clarity. Interviews were based on a cognitive interview methodology using a preplanned questionnaire (see Multimedia Appendix 2) [28]. Focus group participants were asked to (1) read the instructions and tell us what they understood, (2) specify what our questions meant to them, (3) specify what the studied behavior meant to them and what a wiki-based reminder represented to them, (4) identify any ambiguous or complex terms, (5) evaluate their ease or difficulty in answering our questions and examine the difficulties, (6) identify the questions that were the most difficult to understand, (7) specify if each answer option was clearly different from the others and, if not, identify those that were too similar, and (8) suggest changing answer options that were ambiguous or that did not translate their thought processes adequately. We also asked questions about the questionnaire length and consequent participant fatigue. Focus groups were recorded, transcribed verbatim, and then analyzed to identify the adjustments that were needed to the wording of some items and to the visual presentation of the questionnaires.

### Phase 4: Test and Retest

Once adjustments based on the focus groups’ comments were made, a test-retest study of the revised questionnaires was done with a different convenience sample of 25 EPs and 26 AHPs. These participants had not participated in the elicitation phase (Phase 1) or in the focus groups (Phase 2), but worked in the same 3 trauma centers (levels I, II, and III). The EP participants were recruited from these 3 EDs after presenting this project at a monthly departmental meeting in each center. The AHP participants were recruited after contacting the head of each service and asking them to identify potential participants. Consent to participate in this test-retest study was obtained from all participants after explaining the length of the questionnaire and they were informed that all personal information would remain confidential. However, participants were assigned a unique identifier code to write on their questionnaires to link their test and retest responses. This unique identifier was stored separately from the results. The same questionnaire was performed 2 weeks later with the same participants (retest). For this phase, we again allowed participants to choose between the paper-based and the Web-based questionnaire, but they had to use the same modality for both the test and retest. SurveyMonkey automatically collected the data for the Web version, but responses were manually entered in a spreadsheet for the paper-based questionnaires. The Web-based questionnaire did not contain an official completeness check to identify unanswered items; however, participants were allowed to review all their responses by returning to previous items and participants were asked at the last page to complete all items. We monitored duplicate participation with the unique identifier provided.

### Data Analysis

Simple descriptive statistics were used to compare demographic information for EP and AHP participants in our test-retest sample. We also used simple descriptive statistics to compare the demographics of the participants who used the paper-based vs the Web-based questionnaire. We conducted *t* tests for normally distributed continuous variables and Wilcoxon-Mann-Whitney tests for continuous variables that were not normally distributed. For all categorical variables, we used the Fisher exact test. The internal consistency of the constructs (the tendency of answers within a group of constructs) was measured using Cronbach alpha coefficient. To measure stability over time in the constructs, an agreement intraclass correlation coefficient (ICC) was measured. We used criteria published by Landis and Koch to determine the level of consistency and reproducibility of our items [29]. For any missing data for single questionnaire items, we imputed the average of the other items measuring the same construct. Statistical analysis was performed by a biostatistician using SAS version 9.3 (SAS Institute Inc., Cary, NC, USA).

## Results

### Participants

**Figure 1:**
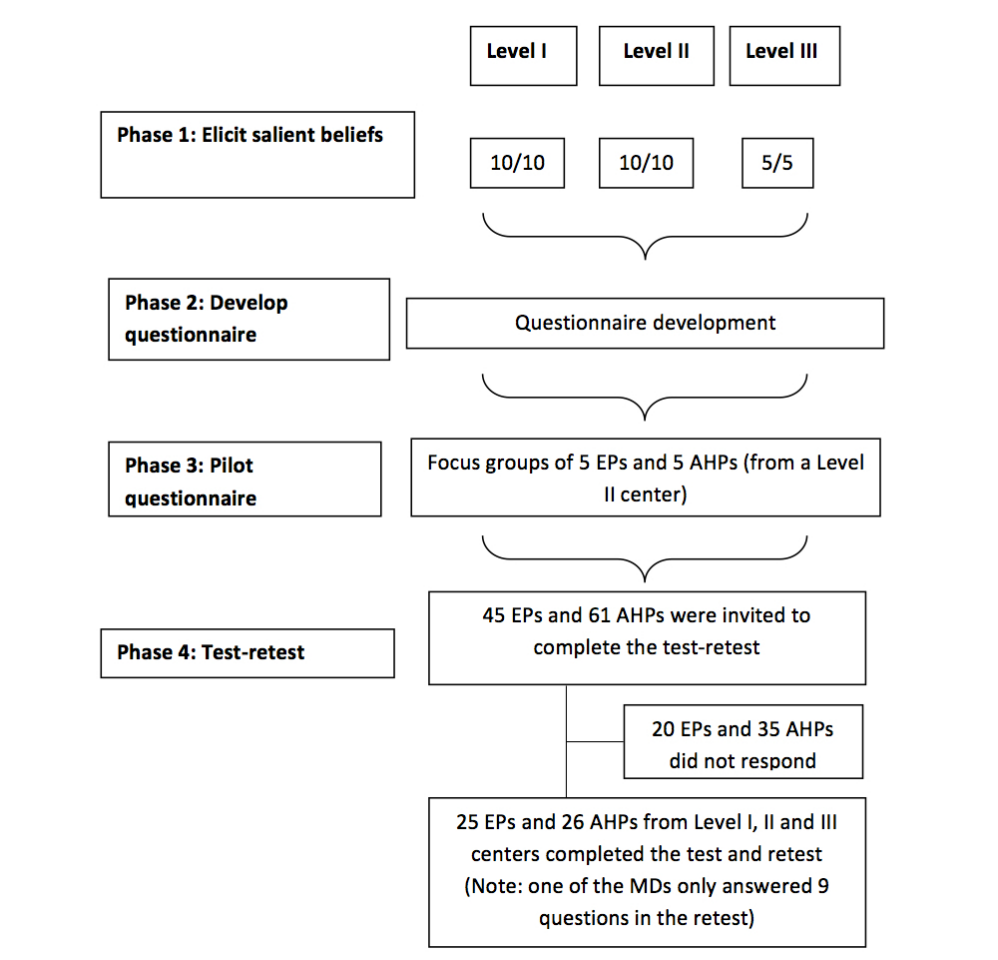
Number of participants per phase of the study. EPs: physicians; AHPs: allied health professionals.

The number of participants per phase of the study is presented in a flow diagram (Figure 1).

Demographic characteristics of the focus groups and test-retest participants are presented in Table 1.

Five EPs and 5 AHPs (3 nurses, 1 respiratory therapist, and 1 pharmacist) formed the focus groups. Among all the test-retest participants, 25% (13/51) came from the level I trauma center, 63% (32/51) from the level II and 12% (6/51) from the level III. Among the 26 test-retest AHPs, there were 12 nurses, 7 respiratory therapists, and 7 pharmacists. Most participating EPs were certified in Emergency Medicine by the College of Family Physicians of Canada (84%, 21/25), 12% (3/25) were certified by the Royal College of Physicians of Canada, and 1 was a family physician certified by the College of Family Physicians of Canada without any Emergency Medicine certification. Compared to the AHPs, the EPs participating in the test-retest were more likely to be older (*P*=.03), male (*P*<.001), to have better access to the Internet (*P*=.02) and to report a higher prevalence of wiki use for personal purposes (72% vs 31%, *P*=.005). Although more EPs tended to use wikis for professional purposes (20%) than AHPs (8%), this difference was not significant. None of our test-retest participants had ever edited a wiki before. However, in our focus groups 1 EP and 1 AHP had edited a wiki.

**Table 1:** Demographic characteristics of the focus groups and test-retest participants.

Variables		Focus groups		Test-retest participants		
		EPs (n=5)	AHPs (n=5)	EPs (n=25)	AHPs (n=26)	*P* value^a^
**Age (years)**						
	Mean (SD)	49 (10)	36 (3)	41.5 (8.9)	35.5 (11.6)	.03
	Median (IQR)	48 (41-56)	36 (35-38)	42 (34-48)	33 (26-43)	
**Gender, n (%)**						
	Male	4 (80)	2 (40)	19 (76)	3 (12)	<.001
	Female	1 (20)	3 (60)	6 (24)	23 (88)	
**Trauma center level, n (%)**						.23
	I	0	0	6 (24)	7 (27)	
	II	5 (100)	5 (100)	18 (72)	14 (54)	
	III	0	0	1 (4)	5 (19)	
**Profession, n (%)**						
	Physician	5 (100)		25 (100)		
	Nurse		3 (60)		12 (46)	
	Respiratory therapist		1 (20)		7 (27)	
	Pharmacist		1 (20)		7 (27)	
**Emergency medicine certification, n (%)**			N/A		N/A	
	College of Family Physicians	2 (40)		21 (84)		
	Royal College of Physicians of Canada	3 (60)		3 (12)		
	Other	0		1 (4)		
**Clinical experience (years)**						
	Mean (SD)	15 (9)	12 (4)	12.5 (9.4)	13.2 (11.1)	.79
	Median (IQR)	13 (8-18)	12 (11-14)	11 (5.5-17)	10.8 (5-19.8)	
Internet access in the ED, n (%)		5 (100)	3 (60)	25 (100%)	20 (77)	.02
Professional use of a wiki, n (%)		2 (40)	2 (40)	5 (20)	2 (8)	.25
Personal use of a wiki, n (%)		4 (80)	2 (40)	18 (72)	8 (31)	.005
Previous editing of a wiki, n (%)		1 (20)	1 (20)	0 (0)	0 (0)	>.99

^a^ *P* values were only calculated for the test-retest group.

### Phase 2: Number and Content of Items in the First Version of the Questionnaires

In the EP questionnaire, there were a total of 13 pages with 63 items: 13 direct construct items, 35 indirect construct items, and 15 demographic characteristic items. For the AHP questionnaire, we created 11 pages with 58 items: 13 direct construct items, 31 indirect construct items, and 14 demographic characteristic items. The original versions of the questionnaires developed during Phase 2 are available in French (see Multimedia Appendices 3 and 4).

### Phase 3: Focus Group Comments and Changes Made to the Questionnaires

In the focus groups, all EPs chose to answer the Web-based survey rather than the paper-based version. The reason evoked was that it was easier to access the survey after receiving the email invitation. One AHP chose the paper version because this participant had not filled out the questionnaire before the focus group and all the other AHPs used the Web-based questionnaire before attending the focus group. Multimedia Appendix 5 lists all the comments made by our focus group participants and the changes we made to the questionnaire in consequence. In summary, we changed the wording of certain items, reduced the length of the questionnaire (without reducing any of the TPB items), removed 1 item from our demographic questions (a question about Wi-Fi availability), and clarified certain items to make the questionnaire easier to complete.

### Phase 4: Test-Retest Results and Changes Made to the Questionnaires

Table 2 compares the characteristics of participants who chose the Web-based questionnaire with those of participants who chose the paper-based questionnaire.

Most EPs (72%, 18/25) used the paper-based version. In contrast, most AHPs (92%, 24/26) preferred the Web-based version for the test-retest and only 2 of 26 AHPs (8%) chose to use the paper-based version. There were no significant differences in characteristics between EPs who used the Web-based vs the paper-based questionnaire. However, 16 of 18 EPs who used the paper-based version did so because the survey was presented during a monthly department meeting when Internet access was not available. Among EPs who had the choice between paper and Web, 6 of 9 (67%) opted for the Web-based questionnaire. The 2 AHPs who chose the paper version worked at the level III trauma center and did not have Internet access in their hospital. All participants used the same administration mode (paper vs Web) for the test and retest. All participants completed the test and retest except 1 EP who only completed 9 items in the retest questionnaire and did not complete it. The data for these 9 items (all relating to direct TPB questionnaire items) were retained in our analysis, but no imputation was performed for the missing data for this participant. There were no duplicate participants.

**Table 2:** Demographic characteristics of participants using Web-based questionnaire vs paper-based questionnaire.

Variables		Emergency physicians (n=25)^a^			Allied health professionals (n=26)		
		Paper (n=18)	Web (n=7)	*P*	Paper (n=2)	Web (n=24)	*P*
**Age (years)**							
	Mean (SD)	41.6 (8.6)	41.3 (10.5)	.95	43.5 (20.5)	34.8 (11.4)	.44
	Median (IQR)	42 (36-47)	41 (32-49)		44 (32-49)	33 (24-35)	
**Gender, n (%)**				.74			.60
	Male	14 (78)	5 (71)		0 (0)	3 (12.5)	
	Female	4 (22)	2 (29)		2 (100)	21 (87.5)	
**Trauma center level, n (%)**				.007			.003
	I	2 (11)	4 (57)		0 (0)	7 (29)	
	II	16 (88)	2 (29)		0 (0)	14 (58)	
	III	0 (0)	1 (14)		2 (100)	3 (12.5)	
**Certification, n (%)**				.11		N/A	
	College of Family Physicians	16 (88)	4 (57)				
	Royal College of Physicians	2 (11)	2 (29)				
	Other	0 (0)	1 (14)				
**Profession, n (%)**							.13
	Physician	18 (100)	7 (100)				
	Nurse				0 (0)	12 (50)	
	Respiratory therapist				2 (100)	5 (20)	
	Pharmacist				0 (0)	7 (29)	
**Clinical experience (years)**							
	Mean (SD)	12.2 (9.1)	13.1 (11.1)	.88	21 (22.6)	12.6 (10.2)	.55
	Median (IQR)	11 (5-18)	11 (5-20)		21 (13-29)	11 (5-17)	
Internet access in ED, n (%)		18 (100)	7 (100)	>.99	0 (0)	20 (83%)	.046
Professional use of a wiki, n (%)		4 (22)	1 (14)	.66	1 (50)	1 (4)	.15
Personal use of a wiki, n (%)		12 (66)	6 (86)	.63	2 (100)	6 (25)	.09
Previous editing of a wiki, n (%)		0 (0)	0 (0)	>.99	0 (0)	0 (0)	>.99

^a^16 EPs were asked to use the paper-based survey and the 9 others could choose Web- or paper-based. All AHPs had the choice to use either Web- or paper-based.

Overall psychometric properties of our 2 questionnaires are presented in Table 3. Internal consistency and temporal stability for each individual item in both questionnaires are presented in Multimedia Appendix 6.

The internal consistency of the items in the EP questionnaire was high (Cronbach alpha >.8) for 4 constructs (intention, attitudinal beliefs, normative beliefs, and control belief facilitators), substantial (Cronbach alpha=.6-.8) for 3 constructs (attitude, perceived behavioral control, and subjective norm), and moderate (Cronbach alpha=.4-.6) for control belief barriers (test Cronbach alpha=58; retest Cronbach alpha=.67). One item measuring the attitude construct was removed due to lack of consistency (test: Cronbach alpha=.06; retest: Cronbach alpha=.02) (see Multimedia Appendix 6). This reduced the length of the questionnaire without affecting the internal consistency of this construct (3 items to measure attitude: test: .89; retest: .91). One item measuring perceived behavioral control was formulated differently because it had low consistency (test: Cronbach alpha=.20; retest: Cronbach alpha=.26) (see Multimedia Appendix 6). For the control beliefs, 2 items were reformulated to increase their internal consistency. All items were considered moderately stable over time (ICC >.4) except for the items measuring facilitators (ICC=.30). Consequently, we reformulated the 3 items concerning facilitators and removed items that represented beliefs that were less salient (based on their ranking in our Phase 1 study) to decrease the questionnaire length.

For the AHP questionnaire, internal consistency was high (Cronbach alpha >.8) for 3 constructs (attitude, attitudinal beliefs, and normative beliefs), substantial (Cronbach alpha=.6-.8) for 2 constructs (intention and control beliefs facilitators), and moderate for 3 constructs (subjective norm, perceived behavioral control, and control beliefs barriers). Therefore, we reformulated items with poor internal consistency: 1 item measuring intention, 1 item for perceived behavioral control, and 1 item for subjective norm. The stability of the items in our AHP questionnaire was good for most constructs except control beliefs facilitators (ICC= –.05). Because the consistency and stability of the items measuring control beliefs (barriers and facilitators) were low, we removed 2 items measuring control beliefs (1 barrier and 1 facilitator) that were not consistent or stable.

The changes made to all the items in our questionnaires are listed in Multimedia Appendices 7 (EPs) and 8 (AHPs) and the final versions (in French) are found in Multimedia Appendices 9 (EPs) and 10 (AHPs). English versions of the questionnaires are also available (Multimedia Appendices 11 and 12). The final EP questionnaire has 11 pages with 55 items and the AHP questionnaire has 9 pages containing 53 items.

**Table 3:** Overall internal consistency (Cronbach alpha) and temporal stability (intraclass correlation coefficient, ICC) of our questionnaire

Questionnaire constructs	Emergency physicians			Allied health professionals		
	Internal consistency		Stability	Internal consistency		Stability
	Test	Retest	ICC	Test	Retest	ICC
Intention	.94	.98	.89	.69	.81	.48^a^
Attitude	.74	.72^b^	.70	.85	.87	.83
Subjective norm	.79	.78	.75	.47^a^	.82	.62
Perceived behavioral control	.65	.67^a^	.68	.55^a^	.62	.60
Attitudinal beliefs	.94	.86	.60	.92	.91	.82
Normative beliefs	.83	.87	.80	.85	.90	.74
Control beliefs-barriers	.58	.67^c^	.78	.58^d^	.55	.66
Control beliefs-facilitators	.97	.85	.30^e^	.72	.94	–.05^f^

^a^1 item was reformulated.

^b^1 item was removed (with 3 items: test Cronbach alpha=.89, retest Cronbach alpha=.91, ICC=.78).

^c^2 items were reformulated.

^d^2 items were removed.

^e^3 items were reformulated and 2 removed.

^f^1 item was removed.

## Discussion

### Principal Results

The objectives of this study were to develop and test the psychometric properties of 2 questionnaires exploring the intention of ED health care professionals and the determinants of this intention to use wiki-based reminders promoting best practices for the management of severe TBI victims. Building on a previous study that had identified the salient beliefs of health care professionals about using wiki-based reminders, our 2 questionnaires will also measure the importance of each of these beliefs. The 4 videos developed in support of these 2 questionnaires helped the participants understand the behavior being investigated. The EP questionnaire now contains 55 items and the AHP questionnaire contains 53 items including the demographic items, as opposed to their original 68 and 58 items, respectively. Both questionnaires take approximately 10 minutes to complete after viewing a 6-minute video. Although some items needed reformulating, our questionnaires now have adequate validity and reliability for large surveys. These results lead us to the following observations.

First, to our knowledge, these questionnaires are among the first to be developed to understand how to implement a wiki that will promote best clinical practices in any area of health care. Other authors have used the Technology Assessment Model to explore how health care professionals use and contribute to social media in general to share medical knowledge with other physicians in the medical community [24]. In contrast, we developed and validated our questionnaires by rigorously following the TPB methodology [30] and included all its constructs, both direct and indirect. As a result, our questionnaires will allow researchers to identify which behavioral determinants are most influential and, therefore, which determinants should be targeted when developing a theory-based intervention.

Second, these 2 questionnaires were developed and validated for 4 types of professionals (EPs, registered nurses, respiratory therapists, and pharmacists) and, thus, are ready for use across this range of health care professionals. The questionnaires are similar in terms of number of items, length of administration, and direct construct items, and many of the items investigating indirect construct items are similar (eg, validity of the information, hospital administration support, ease of access to the wiki-based reminder). However, other items exploring indirect constructs differ from 1 questionnaire to the other depending on the different salient beliefs held by each group of professionals (information captured in Phase 1). For example, the EP questionnaire contains an item investigating the influence of surgeons on their intention to use a wiki-based reminder to care for TBI victims, whereas the AHP questionnaire contains instead an item exploring the influence of quality control managers on their intention. Our results confirm our decision to begin our study by exploring salient beliefs and adapt each questionnaire accordingly. For example, our finding of higher current wiki use and Internet access among EPs than among AHPs supports the need for interventions adapted to each profession. Future investigations using our questionnaires will help us verify the importance of these factors among the different ED health care professionals and then construct profession-specific interventions to guide the implementation of a wiki promoting best practices in TBI trauma care.

Third, some of the constructs in our questionnaires lacked high levels of consistency (eg, perceived behavioral control and control beliefs), more so in the AHP questionnaire than in the EP questionnaire. One explanation for this lack of internal consistency is that the AHP participants were a more heterogeneous group than the EP participants. The AHPs were nurses, respiratory therapists, and pharmacists who all have different clinical tasks and who potentially perceive different levels of control over their clinical practice and behaviors. Moreover, although we tried to make the studied behavior as clear as possible by using profession-specific videos and repeating the description of the behavior in each question, it is still possible that participants did not all have the same behavior in mind. Future investigations with larger samples will help us verify these discrepancies between groups of professionals, and will be important to consider in a future implementation strategy.

Fourth, if given the choice, EPs and AHPs preferred to use the Web-based version rather than the paper version. Although our small sample size prevented us from comparing the internal consistency and stability of the Web-based version compared to the paper-based version, we must continue to have a paper version available because some professionals do not have Internet access (eg, AHPs and those working in smaller trauma centers). Offering a paper version will also allow us to capture the opinions of professionals who are not computer- or Web-savvy and yet are important stakeholders to consider in a future theory-based intervention. Most importantly, lack of Internet access among participants in this survey is an important barrier that must be addressed in any future wiki intervention. For our survey, we addressed this barrier by installing the survey videos on local hospital computers.

Fifth, the videos we created proved to be a useful tool for helping assess the intention of health care professionals to use wiki-based reminders. Using a video was advantageous in that the behavior being studied is new and complex (ie, to use a wiki-based reminder promoting best practice for the management of severe TBI victims in the ED in the Province of Quebec, Canada) and depends on many smaller microbehaviors (eg, logging on to the Internet, using a keyboard to type in the search terms to find the wiki-based reminder, checking off the appropriate prescriptions suggested by the wiki-based reminder). These videos allowed respondents to understand all the small implicit lead-in behaviors necessary to performing the behavior that we were studying.

Finally, EPs and AHPs in our sample reported lower wiki use for professional purposes (20% and 8%) than reported in a recent review of wiki use in health care. This review identified studies reporting a range of usage rates ranging from 18% for nurse practitioners and physician assistants to 35% for pharmacists, 55% for consultants, and 80% for junior physicians [17,31-34]. Although these differences are possibly due to our small convenience sample, future surveys with larger samples will produce better estimates of current wiki use for professional purposes.

### Limitations

Our study has some limitations. First, we had originally planned to use the 2-arm expectancy-value model to measure the influence of indirect constructs (salient beliefs, ie, behavioral beliefs, outcome evaluation, normative beliefs, motivation to comply, control beliefs, and perceived power to influence). However, considering the large number of salient beliefs we retained in our qualitative survey and the fact that the 2-arm expectancy-value model has not shown any advantage over simply using 1 arm of the belief-based measures (only measuring behavioral beliefs, normative beliefs, and control beliefs), we decided to only include items measuring these 3 belief-based measures in our questionnaire [35]. This reduced the number of items in our final questionnaire and likely lowered its administration time, thus reducing participant fatigue and the boredom of answering questions that seem redundant in the 2-arm expectancy model. Shorter questionnaires have been shown to produce more valid information [36].

Second, certain indirect items in our questionnaire lacked temporal stability (eg, control beliefs). This lack of temporal stability might be due to participants changing their assessment of the importance of the different facilitators after 2 weeks, especially if they decided not to watch the video before the retest to save time. Although the retest instructed participants to watch the video again, we did not ask participants if they actually followed this instruction.

Third, our use of the TPB limits our capability to directly assess the importance of environmental factors such as organizational readiness for change. The use of the Theoretical Domains Framework to inform our theory-based intervention could correct this [37,38].

Finally, our questionnaire does not measure the determinants of contributing to the wiki, in addition to consulting it. By definition, a wiki is a product of its users and remains relevant only if its users continue to update it and create new content. Getting experts and other members of a wide community to contribute to a collaborative writing project is a difficult task and a theory-based approach will be needed to stimulate and promote this behavior [14,39,40]. Unfortunately, time constraints are a major barrier when studying clinician behavior in the ED [41,42]. Thus, questionnaire length limited the number of behaviors we could assess in this study. Several further behaviors will need to be studied in the future, but we chose the one we felt to be the most important (using the wiki). If clinicians do not intend to consult a wiki during their clinical duties, we need to understand the determinants of this behavior before asking them to update and create content.

### Future Studies

The next step will be to use these questionnaires in a larger population of ED health care professionals in the Province of Quebec. However, to use this questionnaire in an even broader population across Canada and internationally, our survey instruments (videos and questionnaires) will need to be translated from French to English and other languages and validated using cross-cultural adaptation of the self-report measures [43]. Although we have produced an English version of our questionnaires (Multimedia Appendices 11 and 12), they still need to be validated with a population of English-speaking health care professionals before they can be used in large surveys. Using these questionnaires in multiple settings and countries will help identify the behavioral determinants that a future theory-based intervention should target in order to stimulate the use of wikis promoting best practices in trauma care around the world. Although our questionnaires already contain certain items that are not exclusively relevant to trauma care and EDs and which could serve as a basis for new questionnaires investigating the intention to use wiki-based reminders in other fields of health care, in order to use them to investigate the use of wiki-based reminders in other settings they need to be adapted and validated.

### Conclusion

Our newly developed theory-based questionnaire to measure health care professionals’ intention to use wiki-based reminders has adequate validity and reliability for use in a large survey. In the long run, this will help develop theory-based interventions for wikis promoting best practices in trauma care.

## Multimedia Appendix 1

Links to YouTube videos presenting the behaviour studied.

## Multimedia Appendix 2

Cognitive interview questionnaire (in French).

## Multimedia Appendix 3

AHP questionnaire developed during Phase 2.

## Multimedia Appendix 4

MD questionnaire developed during Phase 2.

## Multimedia Appendix 5

Comments made by focus group participants and changes made to questionnaire.

## Multimedia Appendix 6

Analysis of internal consistency and temporal stability for each item in the MD and AHP questionnaires.

## Multimedia Appendix 7

Changes made to the EP questionnaire after the two-week test-retest.

## Multimedia Appendix 8

Changes made to the AHP questionnaire after the two-week test-retest.

## Multimedia Appendix 9

Final AHP questionnaire (French).

## Multimedia Appendix 10

Final MD questionnaire (French).

## Multimedia Appendix 11

Final AHP questionnaire (English).

## Multimedia Appendix 12

Final MD questionnaire (English).

## Abbreviations

AHP: allied health professional
ED: emergency department
EP: emergency physician
ICC: intraclass correlation coefficient
TBI: traumatic brain injury
TPB: Theory of Planned Behavior
