# Supplementary material for: Development and Validation of Questionnaires Exploring Health Care Professionals' Intention to Use Wiki-Based Reminders to Promote Best Practices in Trauma
Source: JMIR Res Protoc. 2014 Oct 3;3(4):e50. doi: 10.2196/resprot.3762 (PMC4213801; doi:10.2196/resprot.3762)
Supplement: Supplementary file 1 [file resprot_v3i3e50_app1.pdf]

Multimedia Appendix 1:

Links to YouTube™ videos presenting the behavior studied (in French)

Video for physicians: https://www.youtube.com/watch?v=kKCHdm0P8Mg&list=FLGpK694I5Rpw2VRro-yVabQ&index=4

Video for registered nurses: https://www.youtube.com/watch?v=ybHtadfHJJ4&index=3&list=FLGpK694I5Rpw2VRro-yVabQ

Video for respiratory therapists: https://www.youtube.com/watch?v=Z1jmePKwd18&index=2&list=FLGpK694I5Rpw2VRro-yVabQ

Video for pharmacists: https://www.youtube.com/watch?v=dt2AknU5nFo&list=FLGpK694I5Rpw2VRro-yVabQ&index=1
