# Supplementary material for: Development and Validation of Questionnaires Exploring Health Care Professionals' Intention to Use Wiki-Based Reminders to Promote Best Practices in Trauma
Source: JMIR Res Protoc. 2014 Oct 3;3(4):e50. doi: 10.2196/resprot.3762 (PMC4213801; doi:10.2196/resprot.3762)
Supplement: Supplementary file 2 [file resprot_v3i3e50_app2.pdf]

Multimedia Appendix 2: Cognitive interview questionnaire (in French)

Essai pilote, focus group – Entrevue cognitive

Consignes – prévoir 1h

Le but de cette essai pilote est de tester le contenu du questionnaire pour vérifier si les questions sont faciles à comprendre et à répondre. Même si vous allez répondre aux questions, nous sommes intéressés à la façon dont vous avez obtenu les réponses plutôt qu'aux réponses.

***Enregistrer l'entrevue**

1. Demander aux participants de lire **les directives pour répondre** au questionnaire.

a) Dites-moi dans vos mots, comment il faut répondre au questionnaire.

2. Demander aux participants de lire **la définition du l'utilisation du wiki** du questionnaire.

a) Pouvez-vous m'expliquer dans vos propres mots ce que veut dire « un aide-mémoire basé dans un wiki » ?

b) Qu'est-ce que cela veut dire pour vous « d'utiliser un aide-mémoire basé dans un wiki promouvant la meilleure pratique de prise en charge des traumatisés crâniens sévères au département d'urgence au Québec » ?

c) Pouvez-vous me dire d'où provient l'aide-mémoire basé un wiki auquel le questionnaire fait références?

d) Pouvez-vous me donner des exemples d'utilisation de l'aide-mémoire basé dans un wiki?

3. Demander aux participants de **lire et de compléter le questionnaire** et de noter les questions qui semblent moins claire, difficile à comprendre ou à répondre.

a) Parmi les questions que vous avez lues, y en a-t-il qui ne vous ont pas semblées claires, ou qui ont été plus difficiles à répondre?

b) Indiquer le numéro de la ou des questions :

REVOIR CHACUNE DES QUESTIONS IDENTIFIÉES PAR LE RÉPONDANT. POUR CHAQUE QUESTION, DEMANDER : QU'EST-CE QUE VOUS AVEZ OBSERVÉ CONCERNANT CETTE QUESTION?

Si le répondant mentionne que la question n'est pas claire :

c) Pouvez-vous me dire dans vos propres mots ce qui est demandé dans cette question?

Si le répondant mentionne qu'il est difficile de répondre à une question :

d) À quoi avez-vous pensé lorsque vous avez tenté de répondre à cette question?

Avez-vous d'autres commentaires?

Questions spontanées

- À quel point êtes-vous certain de votre réponse?
- La question était-elle difficile ou facile à répondre?
- À quoi pensez-vous lorsque vous répondez?
-

*Reference: Willis, G.B. (2005). *Cognitive interviewing: A tool for improving questionnaire design*. Thousand Oaks : Sage Publications Inc.
