# Supplementary material for: Development and Validation of Questionnaires Exploring Health Care Professionals' Intention to Use Wiki-Based Reminders to Promote Best Practices in Trauma
Source: JMIR Res Protoc. 2014 Oct 3;3(4):e50. doi: 10.2196/resprot.3762 (PMC4213801; doi:10.2196/resprot.3762)
Supplement: Supplementary file 3 [file resprot_v3i3e50_app3.pdf]

Centre de santé et de services sociaux
Alphonse-Desjardins
Centre hospitalier affilié universitaire de Lévis

FCRSS CHSRF
FONDATION CANADIENNE DE LA RECHERCHE SUR LES SERVICES DE SANTÉ
CANADIAN HEALTH SERVICES RESEARCH FOUNDATION

UNIVERSITÉ LAVAL
HÔTEL-DIEU DE LÉVIS
Centre de recherche du
Centre hospitalier affilié universitaire
Hôtel-Dieu de Lévis (CHAU)

ÉTUDE SUR L'UTILISATION DU WIKI

QUESTIONNAIRE

PROJET WIKI (B)

Date : ________________
jj/mm/aaaa

1. Ce questionnaire porte sur votre utilisation d’un aide-mémoire basé dans un wiki promouvant une pratique exemplaire pour la prise en charge des traumatisés crâniens sévères dans les salles d’urgence du Québec.

   Voici la définition de l’utilisation du wiki :

   - Un wiki est un site Web programmé pour permettre l'édition par toute personne ayant accès au site. Il est donc un outil de travail collaboratif. Plus concrètement, dans le monde de la santé, un wiki pourrait permettre aux médecins et autres professionnels de partager et de mettre à jour des aide-mémoire selon les dernières données probantes. Une fois implanté dans un centre hospitalier, il permettrait à tous les professionnels de la santé y ayant accès de le consulter directement sur leurs lieux de travail.
   - **Par exemple,** dans le cas d’un traumatisme crânien sévère, l’aide-mémoire basé dans un wiki pourra être trouvé sur le web à partir d’un ordinateur. Il pourra ensuite être affiché sur un écran et l’équipe pourra l’utiliser pour intervenir.
   - **Veuillez répondre aux questions dans l’éventualité de l’implantation d’un tel aide-mémoire basé dans un wiki** et qu’il a été créé par des collègues de votre centre selon le guide de pratique du *Brain Trauma Foundation* de 2007 et avec les dernières données probantes de la littérature.

2. Bien que certaines questions puissent vous sembler répétitives, il est très important de **répondre à toutes les questions**.

3. Pour répondre aux questions, vous devez inscrire votre réponse à l’endroit reflétant le mieux votre opinion ou votre situation.

Exemple

Il fait très chaud au Québec en été.

				✓		
Très en désaccord	Assez en désaccord	Légèrement en désaccord	Ni en désaccord ni en accord	Légèrement en accord	Assez en accord	Très en accord

4. Notez qu’il n’y a ni bonne ni mauvaise réponse.

5. Vos réponses demeureront confidentielles.

6. Le temps requis pour répondre au questionnaire est d’environ **10 minutes**.

Ce questionnaire porte sur votre opinion concernant l'utilisation potentielle d'un aide-mémoire basé dans un wiki promouvant une pratique exemplaire pour la prise en charge des traumatisés crâniens sévères dans les salles d'urgence du Québec.

1. J'ai l'intention d'utiliser un aide-mémoire basé dans un wiki promouvant une pratique exemplaire pour la prise en charge des traumatisés crâniens sévères dans les salles d'urgence du Québec.

Très en désaccord	Assez en désaccord	Légèrement en désaccord	Ni en désaccord ni en accord	Légèrement en accord	Assez en accord	Très en accord

2. Je vais essayer d'utiliser un aide-mémoire basé dans un wiki promouvant une pratique exemplaire pour la prise en charge des traumatisés crâniens sévères dans les salles d'urgence du Québec.

Très en désaccord	Assez en désaccord	Légèrement en désaccord	Ni en désaccord ni en accord	Légèrement en accord	Assez en accord	Très en accord

3. Je vais utiliser un aide-mémoire basé dans un wiki promouvant une pratique exemplaire pour la prise en charge des traumatisés crâniens sévères dans les salles d'urgence du Québec.

Très improbable	Assez improbable	Légèrement improbable	Ni improbable ni probable	Légèrement probable	Assez probable	Très probable

4. Pour moi, utiliser un aide-mémoire basé dans un wiki promouvant une pratique exemplaire pour la prise en charge des traumatisés crâniens sévères dans les salles d'urgence du Québec serait...

Très difficile	Assez difficile	Légèrement difficile	Ni difficile ni facile	Légèrement facile	Assez facile	Très facile

5. Je me sentirais capable d'utiliser un aide-mémoire basé dans un wiki promouvant une pratique exemplaire pour la prise en charge des traumatisés crâniens sévères dans les salles d'urgence du Québec.

Très en désaccord	Assez en désaccord	Légèrement en désaccord	Ni en désaccord ni en accord	Légèrement en accord	Assez en accord	Très en accord

6. Je suis confiant(e) que je pourrais surmonter les obstacles qui pourraient m'empêcher d'utiliser un aide-mémoire basé dans un wiki promouvant une pratique exemplaire pour la prise en charge des traumatisés crâniens sévères dans les salles d'urgence du Québec.

Très en désaccord	Assez en désaccord	Légèrement en désaccord	Ni en désaccord ni en accord	Légèrement en accord	Assez en accord	Très en accord

7. La plupart des personnes qui sont importantes pour moi me recommanderaient d'utiliser un aide-mémoire basé dans un wiki promouvant une pratique exemplaire pour la prise en charge des traumatisés crâniens sévères dans les salles d'urgence du Québec.

Très en désaccord	Assez en désaccord	Légèrement en désaccord	Ni en désaccord ni en accord	Légèrement en accord	Assez en accord	Très en accord

8. Si j'utilisais un aide-mémoire basé dans un wiki promouvant une pratique exemplaire pour la prise en charge des traumatisés crâniens sévères dans les salles d'urgence du Québec, la plupart des personnes qui sont importantes pour moi seraient...

Très en désaccord	Assez en désaccord	Légèrement en désaccord	Ni en désaccord ni en accord	Légèrement en accord	Assez en accord	Très en accord

9. Les personnes les plus importantes pour moi pensent que je devrais utiliser un aide-mémoire basé dans un wiki promouvant une pratique exemplaire pour la prise en charge des traumatisés crâniens sévères dans les salles d'urgence du Québec.

Très en désaccord	Assez en désaccord	Légèrement en désaccord	Ni en désaccord ni en accord	Légèrement en accord	Assez en accord	Très en accord

10. Pour moi, utiliser un aide-mémoire basé dans un wiki promouvant une pratique exemplaire pour la prise en charge des traumatisés crâniens sévères dans les salles d'urgence du Québec serait...
[Cochez la case appropriée pour ***chacun*** ***des quatre énoncés suivants****]*

a)	☐ Très désagréable	☐ Assez désagréable	☐ Légèrement désagréable	☐ Ni désagréable ni agréable	☐ Légèrement agréable	☐ Assez agréable	☐ Très agréable
b)	☐ Très stressant	☐ Assez stressant	☐ Légèrement stressant	☐ Ni stressant ni relaxant	☐ Légèrement relaxant	☐ Assez relaxant	☐ Très relaxant
c)	☐ Très inutile	☐ Assez inutile	☐ Légèrement inutile	☐ Ni inutile ni utile	☐ Légèrement utile	☐ Assez utile	☐ Très utile
d)	☐ Très insatisfaisant	☐ Assez insatisfaisant	☐ Légèrement insatisfaisant	☐ Ni insatisfaisant ni satisfaisant	☐ Légèrement satisfaisant	☐ Assez satisfaisant	☐ Très satisfaisant

11. Il serait **plus facile** pour moi d'utiliser un aide-mémoire basé dans un wiki promouvant une pratique exemplaire pour la prise en charge des traumatisés crâniens sévères dans les salles d'urgence du Québec... *[Cochez la case appropriée pour* ***chacun*** ***des huit énoncés suivants****]*

	**Très en désaccord**	**Assez en désaccord**	**Légèrement en désaccord**	**Ni en désaccord ni en accord**	**Légèrement en accord**	**Assez en accord**	**Très en accord**
a) **si** l'ordinateur était accessible au chevet du patient.							
b) **si** la qualité scientifique de l'information était validée.							
c) **si** je pouvais l'expérimenter avant de l'utiliser.							
d) **s'il** était facile à utiliser (e.g. la navigation, la recherche d'information).							
e) **s'il** y avait de la publicité sur l'aide-mémoire basé dans un wiki.							
f) **si** le site était sécurisé.							
g) **s'il** y avait un poste de travail pour chaque profession.							
h) **s'il** avait une conception visuelle de qualité.							

12. Je me **sentirais capable** d'utiliser un aide-mémoire basé dans un wiki promouvant une pratique exemplaire pour la prise en charge des traumatisés crâniens sévères dans les salles d'urgence du Québec…
[Cochez la case appropriée pour ***chacun des six énoncés suivants****]*

	Très en désaccord	Assez en désaccord	Légèrement en désaccord	Ni en désaccord ni en accord	Légèrement en accord	Assez en accord	Très en accord
a) **même si** j'ignorais qui détient la responsabilité légale.							
b) **même si** j'avais des contraintes de temps.							
c) **même si** le système informatique n'était pas toujours fiable.							
d) **même si** ce n'est pas toute l'équipe qui est à jour concernant la dernière version de l'aide-mémoire.							
e) **même si** le coût des ordinateurs était élevé.							
f) **même si** son utilisation était obligatoire.							

13. Les médecins de mon centre hospitalier approuveraient que j'utilise un aide-mémoire basé dans un wiki promouvant une pratique exemplaire pour la prise en charge des traumatisés crâniens sévères dans les salles d'urgence du Québec.

Très en désaccord	Assez en désaccord	Légèrement en désaccord	Ni en désaccord ni en accord	Légèrement en accord	Assez en accord	Très en accord

14. Les inhalothérapeutes de mon centre hospitalier approuveraient que j'utilise un aide-mémoire basé dans un wiki promouvant une pratique exemplaire pour la prise en charge des traumatisés crâniens sévères dans les salles d'urgence du Québec.

Très en désaccord	Assez en désaccord	Légèrement en désaccord	Ni en désaccord ni en accord	Légèrement en accord	Assez en accord	Très en accord

15. Le personnel infirmier de mon centre hospitalier approuverait que j'utilise un aide-mémoire basé dans un wiki promouvant une pratique exemplaire pour la prise en charge des traumatisés crâniens sévères dans les salles d'urgence du Québec.

Très en désaccord	Assez en désaccord	Légèrement en désaccord	Ni en désaccord ni en accord	Légèrement en accord	Assez en accord	Très en accord

16. La génération plus jeune d'employés de mon centre hospitalier approuverait que j'utilise un aide-mémoire basé dans un wiki promouvant une pratique exemplaire pour la prise en charge des traumatisés crâniens sévères dans les salles d'urgence du Québec.

Très en désaccord	Assez en désaccord	Légèrement en désaccord	Ni en désaccord ni en accord	Légèrement en accord	Assez en accord	Très en accord

17. Les personnes de mon centre hospitalier moins à l'aise avec l'informatique approuveraient que j'utilise un aide-mémoire basé dans un wiki promouvant une pratique exemplaire pour la prise en charge des traumatisés crâniens sévères dans les salles d'urgence du Québec.

Très en désaccord	Assez en désaccord	Légèrement en désaccord	Ni en désaccord ni en accord	Légèrement en accord	Assez en accord	Très en accord

18. Les personnes de mon centre hospitalier réticentes au changement approuveraient que j'utilise un aide-mémoire basé dans un wiki promouvant une pratique exemplaire pour la prise en charge des traumatisés crâniens sévères dans les salles d'urgence du Québec.

Très en désaccord	Assez en désaccord	Légèrement en désaccord	Ni en désaccord ni en accord	Légèrement en accord	Assez en accord	Très en accord

19. L'équipe de traumatologie de mon centre hospitalier approuverait que j'utilise un aide-mémoire basé dans un wiki promouvant une pratique exemplaire pour la prise en charge des traumatisés crâniens sévères dans les salles d'urgence du Québec.

Très en désaccord	Assez en désaccord	Légèrement en désaccord	Ni en désaccord ni en accord	Légèrement en accord	Assez en accord	Très en accord

20. <u>Les personnes responsables de la qualité des soins de mon centre hospitalier</u> approuveraient que j'utilise un aide-mémoire basé dans un wiki promouvant une pratique exemplaire pour la prise en charge des traumatisés crâniens sévères dans les salles d'urgence du Québec.

Très en désaccord	Assez en désaccord	Légèrement en désaccord	Ni en désaccord ni en accord	Légèrement en accord	Assez en accord	Très en accord

21. <u>L'administration de mon centre hospitalier</u> approuverait que j'utilise un aide-mémoire basé dans un wiki promouvant une pratique exemplaire pour la prise en charge des traumatisés crâniens sévères dans les salles d'urgence du Québec.

Très en désaccord	Assez en désaccord	Légèrement en désaccord	Ni en désaccord ni en accord	Légèrement en accord	Assez en accord	Très en accord

22. Si j'utilisais un aide-mémoire basé dans un wiki promouvant une pratique exemplaire pour la prise en charge des traumatisés crâniens sévères dans les salles d'urgence du Québec, cela...
[Cochez la case appropriée pour ***<u>chacun</u>*** ***des huit énoncés suivants****]*

	**Très en désaccord**	**Assez en désaccord**	**Légèrement en désaccord**	**Ni en désaccord ni en accord**	**Légèrement en accord**	**Assez en accord**	**Très en accord**
a) augmenterait la rapidité d'accès aux aide-mémoire.							
b) améliorerait la qualité des soins que je donne.							
c) me permettrait d'avoir des aide-mémoire mis à jour régulièrement.							
d) uniformiserait les pratiques.							
e) favoriserait le travail d'équipe.							
f) centraliserait l'information et les protocoles.							
g) me donnerait accès aux données probantes.							
h) servirait d'outil pour l'enseignement.							

Données sociodémographiques

23. Quel âge avez-vous? ______ ans

24. Quel est votre sexe?
- ☐ Femme
- ☐ Homme

25. Quelle est votre profession?
- ☐ Infirmier(ère)
- ☐ Inhalothérapeute
- ☐ Pharmacien(ne)
- ☐ Autre________________________________

26. Dans quel type de centre hospitalier travaillez-vous?

☐ **Tertiaire**

Définition : Le centre de traumatologie tertiaire (Level I dans la littérature anglo-saxonne) offre l'éventail complet de soins en trauma, y compris la neurochirurgie, la chirurgie générale et la chirurgie orthopédique. Ce type de centre dirige une unité de soins intensifs dotée d'une équipe d'intensivistes certifiés à temps plein et un département d'urgence doté de médecins d'urgence certifiés par le Collège royal des médecins et chirurgiens du Canada, le Collège des médecins de famille du Canada ou le Collège des médecins du Québec.

☐ **Secondaire**

Définition : Le centre de traumatologie secondaire (Level II dans la littérature anglo-saxonne) offre des soins de chirurgie générale et de chirurgie orthopédique à temps plein et toute l'année. Ce type de centre dirige une unité de soins intensifs dotée d'une équipe d'intensivistes certifiés à temps plein et un département d'urgence doté de médecins d'urgence certifiés.

☐ **Primaire**

Définition : Le centre de traumatologie primaire (Level III dans la littérature anglo-saxonne) offre les soins de chirurgie générale à temps plein et toute l'année ainsi que la couverture partielle de la chirurgie orthopédique. Ce type de centre dirige un département d'urgence doté de médecins généralistes et une unité de soins intensifs qui n'est toutefois pas dotée d'intensivistes certifiés à temps plein.

27. Depuis combien d'années exercez-vous votre métier (après l'obtention de votre diplôme)?

____

28. a) Est-ce qu'il y a un ordinateur avec accès à Internet dans votre salle d'urgence?

☐ Oui
☐ Non

b) Si oui, dans la salle de réanimation?

☐ Oui
☐ Non

c) Avez-vous accès à un réseau wifi (sans fil) fonctionnel accessible au personnel?

☐ Oui
☐ Non

d) Avez-vous accès à un réseau wifi (sans fil) fonctionnel accessible aux patients?

☐ Oui
☐ Non

29. Utilisez-vous actuellement un wiki pour un usage <u>professionnel</u> (e.g., Wikipédia)?

☐ Oui → <u>Lequel</u>? ________________________________
☐ Non

30. Utilisez-vous actuellement un wiki pour un usage <u>personnel</u> (e.g., Wikipédia)?

☐ Oui → <u>Lequel</u>? ________________________________
☐ Non

31. À quelle fréquence consulté vous un wiki pour un usage <u>professionnel</u>?

☐ Rarement
☐ 1 fois par mois
☐ 1 fois par semaine
☐ 3 fois par semaine
☐ Quotidiennement
☐ Plusieurs fois par jour

32. À quelle fréquence consulté vous un wiki pour un usage personnel?

- ☐ Rarement
- ☐ 1 fois par mois
- ☐ 1 fois par semaine
- ☐ 3 fois par semaine
- ☐ Quotidiennement
- ☐ Plusieurs fois par jour

33. Avez-vous édité, par le passé, un wiki?

- ☐ Oui → Lequel? ______________________________
- ☐ Non

34. Êtes-vous membre d'un comité de traumatologie (local ou régional)?

- ☐ Oui
- ☐ Non

Le questionnaire est terminé!

S'il vous plaît, assurez-vous d'avoir répondu à toutes les questions.

MERCI DE VOTRE PRÉCIEUSE COLLABORATION

Si vous avez des commentaires ou suggestions concernant ce questionnaire ou cette recherche, vous pouvez les inscrire ci-dessous.

COMMENTAIRES
