# Supplementary material for: Development and Validation of Questionnaires Exploring Health Care Professionals' Intention to Use Wiki-Based Reminders to Promote Best Practices in Trauma
Source: JMIR Res Protoc. 2014 Oct 3;3(4):e50. doi: 10.2196/resprot.3762 (PMC4213801; doi:10.2196/resprot.3762)
Supplement: Supplementary file 4 [file resprot_v3i3e50_app4.pdf]

Centre de santé et de services sociaux
Alphonse-Desjardins
Centre hospitalier affilié universitaire de Lévis

FCRSS CHSRF
FONDATION CANADIENNE DE LA RECHERCHE SUR LES SERVICES DE SANTÉ
CANADIAN HEALTH SERVICES RESEARCH FOUNDATION

UNIVERSITÉ LAVAL
HÔTEL-DIEU DE LÉVIS
Centre de recherche du
Centre hospitalier affilié universitaire
Hôtel-Dieu de Lévis (CHAU)

ÉTUDE SUR L'UTILISATION DU WIKI

QUESTIONNAIRE

PROJET WIKI (A)

Date : ________________

jj/mm/aaaa

1. Ce questionnaire porte sur votre utilisation d'un aide-mémoire basé dans un wiki promouvant une pratique exemplaire pour la prise en charge des traumatisés crâniens sévères dans les salles d'urgence du Québec.

   Voici la définition de l'utilisation du wiki :

   - Un wiki est un site Web programmé pour permettre l'édition par toute personne ayant accès au site. Il est donc un outil de travail collaboratif. Plus concrètement, dans le monde de la santé, un wiki pourrait permettre aux médecins et autres professionnels de partager et de mettre à jour des aide-mémoire selon les dernières données probantes. Une fois implanté dans un centre hospitalier, il permettrait à tous les professionnels de la santé y ayant accès de le consulter directement sur leurs lieux de travail.
   - **Par exemple,** dans le cas d'un traumatisme crânien sévère, l'aide-mémoire basé dans un wiki pourra être trouvé sur le web à partir d'un ordinateur. Il pourra ensuite être affiché sur un écran et l'équipe pourra l'utiliser pour intervenir.
   - **Veuillez répondre aux questions dans l'éventualité de l'implantation d'un tel aide-mémoire basé dans un wiki** et qu'il a été créé par des collègues de votre centre selon le guide de pratique du *Brain Trauma Foundation* de 2007 et avec les dernières données probantes de la littérature.

2. Bien que certaines questions puissent vous sembler répétitives, il est très important de **répondre à toutes les questions**.

3. Pour répondre aux questions, vous devez inscrire votre réponse à l'endroit reflétant le mieux votre opinion ou votre situation.

Exemple

Il fait très chaud au Québec en été.

				✓		
Très en désaccord	Assez en désaccord	Légèrement en désaccord	Ni en désaccord ni en accord	Légèrement en accord	Assez en accord	Très en accord

4. Notez qu'il n'y a ni bonne ni mauvaise réponse.

5. Vos réponses demeureront confidentielles.

6. Le temps requis pour répondre au questionnaire est d'environ **10 minutes**.

Ce questionnaire porte sur votre opinion concernant l'utilisation potentielle d'un aide-mémoire basé dans un wiki promouvant une pratique exemplaire pour la prise en charge des traumatisés crâniens sévères dans les salles d'urgence du Québec.

1. J'ai l'intention d'utiliser un aide-mémoire basé dans un wiki promouvant une pratique exemplaire pour la prise en charge des traumatisés crâniens sévères dans les salles d'urgence du Québec.

Très en désaccord	Assez en désaccord	Légèrement en désaccord	Ni en désaccord ni en accord	Légèrement en accord	Assez en accord	Très en accord

2. Je vais essayer d'utiliser un aide-mémoire basé dans un wiki promouvant une pratique exemplaire pour la prise en charge des traumatisés crâniens sévères dans les salles d'urgence du Québec.

Très en désaccord	Assez en désaccord	Légèrement en désaccord	Ni en désaccord ni en accord	Légèrement en accord	Assez en accord	Très en accord

3. Je vais utiliser un aide-mémoire basé dans un wiki promouvant une pratique exemplaire pour la prise en charge des traumatisés crâniens sévères dans les salles d'urgence du Québec.

Très improbable	Assez improbable	Légèrement improbable	Ni improbable ni probable	Légèrement probable	Assez probable	Très probable

4. Pour moi, utiliser un aide-mémoire basé dans un wiki promouvant une pratique exemplaire pour la prise en charge des traumatisés crâniens sévères dans les salles d'urgence du Québec serait...

Très difficile	Assez difficile	Légèrement difficile	Ni difficile ni facile	Légèrement facile	Assez facile	Très facile

5. Je me sentirais capable d'utiliser un aide-mémoire basé dans un wiki promouvant une pratique exemplaire pour la prise en charge des traumatisés crâniens sévères dans les salles d'urgence du Québec.

Très en désaccord	Assez en désaccord	Légèrement en désaccord	Ni en désaccord ni en accord	Légèrement en accord	Assez en accord	Très en accord

6. Je suis confiant(e) que je pourrais surmonter les obstacles qui pourraient m'empêcher d'utiliser un aide-mémoire basé dans un wiki promouvant une pratique exemplaire pour la prise en charge des traumatisés crâniens sévères dans les salles d'urgence du Québec.

Très en désaccord	Assez en désaccord	Légèrement en désaccord	Ni en désaccord ni en accord	Légèrement en accord	Assez en accord	Très en accord

7. La plupart des personnes qui sont importantes pour moi me recommanderaient d'utiliser un aide-mémoire basé dans un wiki promouvant une pratique exemplaire pour la prise en charge des traumatisés crâniens sévères dans les salles d'urgence du Québec.

Très en désaccord	Assez en désaccord	Légèrement en désaccord	Ni en désaccord ni en accord	Légèrement en accord	Assez en accord	Très en accord

8. Si j'utilisais un aide-mémoire basé dans un wiki promouvant une pratique exemplaire pour la prise en charge des traumatisés crâniens sévères dans les salles d'urgence du Québec, la plupart des personnes qui sont importantes pour moi seraient...

Très en désaccord	Assez en désaccord	Légèrement en désaccord	Ni en désaccord ni en accord	Légèrement en accord	Assez en accord	Très en accord

9. Les personnes les plus importantes pour moi pensent que je devrais utiliser un aide-mémoire basé dans un wiki promouvant une pratique exemplaire pour la prise en charge des traumatisés crâniens sévères dans les salles d'urgence du Québec.

Très en désaccord	Assez en désaccord	Légèrement en désaccord	Ni en désaccord ni en accord	Légèrement en accord	Assez en accord	Très en accord

10. Pour moi, utiliser un aide-mémoire basé dans un wiki promouvant une pratique exemplaire pour la prise en charge des traumatisés crâniens sévères dans les salles d'urgence du Québec serait...
[Cochez la case appropriée pour ***chacun*** ***des quatre énoncés suivants****]*

a)

Très désagréable	Assez désagréable	Légèrement désagréable	Ni désagréable ni agréable	Légèrement agréable	Assez agréable	Très agréable

c)

Très stressant	Assez stressant	Légèrement stressant	Ni stressant ni relaxant	Légèrement relaxant	Assez relaxant	Très relaxant

d)

Très inutile	Assez inutile	Légèrement inutile	Ni inutile ni utile	Légèrement utile	Assez utile	Très utile

e)

Très insatisfaisant	Assez insatisfaisant	Légèrement insatisfaisant	Ni insatisfaisant ni satisfaisant	Légèrement satisfaisant	Assez satisfaisant	Très satisfaisant

11. Le personnel infirmier de mon centre hospitalier approuverait que j'utilise un aide-mémoire basé dans un wiki promouvant une pratique exemplaire pour la prise en charge des traumatisés crâniens sévères dans les salles d'urgence du Québec

Très en désaccord	Assez en désaccord	Légèrement en désaccord	Ni en désaccord ni en accord	Légèrement en accord	Assez en accord	Très en accord

12. Les médecins de mon centre hospitalier approuveraient que j'utilise un aide-mémoire basé dans un wiki promouvant une pratique exemplaire pour la prise en charge des traumatisés crâniens sévères dans les salles d'urgence du Québec.

Très en désaccord	Assez en désaccord	Légèrement en désaccord	Ni en désaccord ni en accord	Légèrement en accord	Assez en accord	Très en accord

13. Le personnel hospitalier des centres éloignés, moins exposés aux traumatismés crâniens sévères, approuveraient que j'utilise un aide-mémoire basé dans un wiki promouvant une pratique exemplaire pour la prise en charge des traumatisés crâniens sévères dans les salles d'urgence du Québec.

Très en désaccord	Assez en désaccord	Légèrement en désaccord	Ni en désaccord ni en accord	Légèrement en accord	Assez en accord	Très en accord

14. La génération plus jeune d’employés de mon centre hospitalier approuverait que j’utilise un aide-mémoire basé dans un wiki promouvant une pratique exemplaire pour la prise en charge des traumatisés crâniens sévères dans les salles d’urgence du Québec.

Très en désaccord	Assez en désaccord	Légèrement en désaccord	Ni en désaccord ni en accord	Légèrement en accord	Assez en accord	Très en accord

15. Les personnes de mon centre hospitalier qui s’opposent aux soins standardisés approuveraient que j’utilise un aide-mémoire basé dans un wiki promouvant une pratique exemplaire pour la prise en charge des traumatisés crâniens sévères dans les salles d’urgence du Québec.

Très en désaccord	Assez en désaccord	Légèrement en désaccord	Ni en désaccord ni en accord	Légèrement en accord	Assez en accord	Très en accord

16. Les personnes de mon centre hospitalier moins à l’aise avec l’informatique approuveraient que j’utilise un aide-mémoire basé dans un wiki promouvant une pratique exemplaire pour la prise en charge des traumatisés crâniens sévères dans les salles d’urgence du Québec.

Très en désaccord	Assez en désaccord	Légèrement en désaccord	Ni en désaccord ni en accord	Légèrement en accord	Assez en accord	Très en accord

17. Les inhalothérapeutes de mon centre hospitalier approuveraient que j’utilise un aide-mémoire basé dans un wiki promouvant une pratique exemplaire pour la prise en charge des traumatisés crâniens sévères dans les salles d’urgence du Québec.

Très en désaccord	Assez en désaccord	Légèrement en désaccord	Ni en désaccord ni en accord	Légèrement en accord	Assez en accord	Très en accord

18. L’équipe de traumatologie de mon centre hospitalier approuverait que un aide-mémoire basé dans un wiki promouvant une pratique exemplaire pour la prise en charge des traumatisés crâniens sévères dans les salles d’urgence du Québec.

Très en désaccord	Assez en désaccord	Légèrement en désaccord	Ni en désaccord ni en accord	Légèrement en accord	Assez en accord	Très en accord

19. L’administration de mon centre hospitalier approuverait que j’utilise un aide-mémoire basé dans un wiki promouvant une pratique exemplaire pour la prise en charge des traumatisés crâniens sévères dans les salles d’urgence du Québec.

Très en désaccord	Assez en désaccord	Légèrement en désaccord	Ni en désaccord ni en accord	Légèrement en accord	Assez en accord	Très en accord

20. Mes patients approuveraient que j’utilise un aide-mémoire basé dans un wiki promouvant une pratique exemplaire pour la prise en charge des traumatisés crâniens sévères dans les salles d’urgence du Québec.

Très en désaccord	Assez en désaccord	Légèrement en désaccord	Ni en désaccord ni en accord	Légèrement en accord	Assez en accord	Très en accord

21. Les médecins spécialistes (intensivistes, chirurgiens) de mon centre hospitalier approuveraient que j’utilise un aide-mémoire basé dans un wiki promouvant une pratique exemplaire pour la prise en charge des traumatisés crâniens sévères dans les salles d’urgence du Québec.

Très en désaccord	Assez en désaccord	Légèrement en désaccord	Ni en désaccord ni en accord	Légèrement en accord	Assez en accord	Très en accord

22. Il serait **plus facile** pour moi d'utiliser un aide-mémoire basé dans un wiki promouvant une pratique exemplaire pour la prise en charge des traumatisés crâniens sévères dans les salles d'urgence du Québec... *[Cochez la case appropriée pour* ***chacun*** ***des onze énoncés suivants****]*

	**Très en désaccord**	**Assez en désaccord**	**Légèrement en désaccord**	**Ni en désaccord ni en accord**	**Légèrement en accord**	**Assez en accord**	**Très en accord**
a) **s'il** était facile à utiliser (e.g., la navigation et la recherche d'information).							
b) **si** l'ordinateur était accessible au chevet du patient.							
c) **si** la qualité scientifique de l'information était validée.							
d) **si** l'accès à l'aide-mémoire était rapide.							
e) **s'il** n'y avait pas de contrôle institutionnel sur l'accès (e.g., mots de passe, sites bloqués).							
f) **s'il** était compatible avec mon processus de travail.							
g) **s'il** était accessible par appareils de poche (e.g. iPod, iPhone, iPad, Blackberry).							
h) **s'il** était facile de l'adapter à la réalité de mon milieu de travail.							
i) **si** je pouvais l'expérimenter avant de l'utiliser.							
j) **s'il** y avait un accès Internet à mon centre hospitalier.							
k) **s'il** avait une conception visuelle de qualité.							

23. Je me **sentirais capable** d'utiliser un aide-mémoire basé dans un wiki promouvant une pratique exemplaire pour la prise en charge des traumatisés crâniens sévères dans les salles d'urgence du Québec... *[Cochez la case appropriée pour* ***chacun*** ***des cinq énoncés suivants****]*

	**Très en désaccord**	**Assez en désaccord**	**Légèrement en désaccord**	**Ni en désaccord ni en accord**	**Légèrement en accord**	**Assez en accord**	**Très en accord**
a) **même si** l'information n'était pas mise à jour régulièrement.							
b) **même si** j'avais des contraintes de temps.							
c) **même si** l'information changeait fréquemment.							
d) **même si** les auteurs n'étaient pas identifiés.							
e) **même si** j'ignorais qui détient la responsabilité légale.							

24. Si j'utilisais un aide-mémoire basé dans un wiki promouvant une pratique exemplaire pour la prise en charge des traumatisés crâniens sévères dans les salles d'urgence du Québec, cela...
[Cochez la case appropriée pour ***chacun*** ***des huit énoncés suivants****]*

	**Très en désaccord**	**Assez en désaccord**	**Légèrement en désaccord**	**Ni en désaccord ni en accord**	**Légèrement en accord**	**Assez en accord**	**Très en accord**
a) me permettrait de me rafraîchir la mémoire.							
b) me donnerait accès aux données probantes.							
c) permettrait le partage d'information avec d'autres centres hospitaliers.							
d) uniformiserait les pratiques.							
e) centraliserait l'information et les protocoles.							
f) réduirait les erreurs au niveau des interventions.							
g) permettrait d'avoir l'opinion des experts.							
h) augmenterait mon niveau de stress.							

Données sociodémographiques

25. Quel âge avez-vous? ______ ans

26. Quel est votre sexe?
- ☐ Femme
- ☐ Homme

27. Quel est votre profession?
- ☐ Médecin
- ☐ Autre ________________________________

28. Avez-vous une formation en médecine d’urgence?

OUI ☐→ ☐ Collège des médecins de famille du Canada (CMFC)

☐ Collège royal des médecins et chirurgiens du Canada (CRMCC)

NON ☐→ ☐ Autre _______________________________

29. Dans quel type de centre hospitalier travaillez-vous?

☐ **Tertiaire**

Définition : Le centre de traumatologie tertiaire (Level I dans la littérature anglo-saxonne) offre l’éventail complet de soins en trauma, y compris la neurochirurgie, la chirurgie générale et la chirurgie orthopédique. Ce type de centre dirige une unité de soins intensifs dotée d’une équipe d’intensivistes certifiés à temps plein et un département d’urgence doté de médecins d’urgence certifiés par le Collège royal des médecins et chirurgiens du Canada, le Collège des médecins de famille du Canada ou le Collège des médecins du Québec.

☐ **Secondaire**

Définition : Le centre de traumatologie secondaire (Level II dans la littérature anglo-saxonne) offre des soins de chirurgie générale et de chirurgie orthopédique à temps plein et toute l’année. Ce type de centre dirige une unité de soins intensifs dotée d’une équipe d’intensivistes certifiés à temps plein et un département d’urgence doté de médecins d’urgence certifiés.

☐ **Primaire**

Définition : Le centre de traumatologie primaire (Level III dans la littérature anglo-saxonne) offre les soins de chirurgie générale à temps plein et toute l’année ainsi que la couverture partielle de la chirurgie orthopédique. Ce type de centre dirige un département d’urgence doté de médecins généralistes et une unité de soins intensifs qui n’est toutefois pas dotée d’intensivistes certifiés à temps plein.

30. a) Depuis combien d’années exercez-vous votre métier (après la résidence)?

____

31. a) Est-ce qu’il y a un ordinateur avec accès à Internet dans votre salle d’urgence?

- ☐ Oui
- ☐ Non

b) Si oui, dans la salle de réanimation?

- ☐ Oui
- ☐ Non

c) Avez-vous accès à un réseau wifi (sans fil) fonctionnel accessible au personnel?

- ☐ Oui
- ☐ Non

d) Avez-vous accès à un réseau wifi (sans fil) fonctionnel accessible aux patients?

- ☐ Oui
- ☐ Non

32. Utilisez-vous actuellement un wiki pour un usage <u>professionnel</u> (e.g., Wikipédia)?

- ☐ Oui → <u>Lequel</u>? ________________________________
- ☐ Non

33. Utilisez-vous actuellement un wiki pour un usage <u>personnel</u> (e.g., Wikipédia)?

- ☐ Oui → <u>Lequel</u>? ________________________________
- ☐ Non

34. À quelle fréquence consultez-vous un wiki pour un usage <u>professionnel</u>?

- ☐ Rarement
- ☐ 1 fois par mois
- ☐ 1 fois par semaine
- ☐ 3 fois par semaine
- ☐ Quotidiennement
- ☐ Plusieurs fois par jour

35. À quelle fréquence consultez-vous un wiki pour un usage <u>personnel</u>?

- ☐ Rarement
- ☐ 1 fois par mois
- ☐ 1 fois par semaine
- ☐ 3 fois par semaine

☐ Quotidiennement
☐ Plusieurs fois par jour

36. Avez-vous édité, par le passé, un wiki?
☐ Oui → Lequel? ______________________________
☐ Non

37. Êtes-vous membre d'un comité de traumatologie (local ou régional)?
☐ Oui
☐ Non

Le questionnaire est terminé!

S'il vous plaît, assurez-vous d'avoir répondu à toutes les questions.

MERCI DE VOTRE PRÉCIEUSE COLLABORATION

Si vous avez des commentaires ou suggestions concernant ce questionnaire ou cette recherche, vous pouvez les inscrire ci-dessous.

COMMENTAIRES

____
____
____
____
____
____
____
