# Supplementary material for: Development and Validation of Questionnaires Exploring Health Care Professionals' Intention to Use Wiki-Based Reminders to Promote Best Practices in Trauma
Source: JMIR Res Protoc. 2014 Oct 3;3(4):e50. doi: 10.2196/resprot.3762 (PMC4213801; doi:10.2196/resprot.3762)
Supplement: Supplementary file 5 [file resprot_v3i3e50_app5.pdf]

Multimedia Appendix 5: Comments made by focus group participants and changes made to questionnaire

Suggestions by participants	Changes (or justification for not making changes) to the questionnaire
In the description of the behavior, the use of "promouvant" needs to be changed to "qui promeut".	" (...) l'utilisation potentielle d'un aide-mémoire basé dans un wiki **promouvant une pratique** exemplaire (...)" This sentence was changed to: "(...) l'utilisation potentielle d'un aide-mémoire basé dans un wiki **qui promeut** une pratique exemplaire (...)"
Most participants stated that it took them 6 minutes to view the video and 10-12 minutes to answer the questionnaire. Although there were mixed feelings about the length of the questionnaire, most participants felt that the questionnaire length should be reduced.	1- We combined the questions relating to normative beliefs that had been separated into individual questions in the first draft into one question containing a single introduction with multiple sub-items. In the EP questionnaire, we combined questions #11 to 21 into a single question #12 with multiple sub-items for grading. In the AHP questionnaire, we replaced questions #13 to 21 with a single question (#12) with multiple sub-items.<br>2- In the demographic characteristics section: **1)** we removed the definitions for the different levels of trauma center; **2)** we removed the question concerning the availability of WIFI for professionals and/or for patients, as we did not believe the question added much to our survey results; and **3)** we combined the questions on self-reported frequency of using/editing other wikis into a single open question so that it would not take as long to read.<br>3- The original research process involved a separate consent form that participants had to sign. The ethics review board accepted that we simply include a simple consent statement at the beginning of our questionnaire that participants had to check off before continuing the questionnaire.<br>4- For the web-based SurveyMonkey version, the ethics review board asked us to add a statement notifying participants that due to the U.S. Patriot Act, their answers could be audited by the U.S. government for anti-terrorist safety reasons.
Questions seem very similar. In particular, questions 1, 2 and 3 (questions concerning the intention construct) seem repetitive.	We decided to randomly mix all the items for the different constructs so that items measuring similar constructs would not be grouped together. However, we did keep questions on the indirect constructs concerning advantages, disadvantages, barriers, facilitators, and positive and negative referents grouped together to reduce the length of our questionnaire.
The sentences describing each item seem long.	As the behavior being studied is repeated in each item, we bolded the parts of each question that changed from one question to another and which we wanted the participants to focus on.
The web-based questionnaire contained a spelling mistake.	The spelling mistake in the word "désagréable" was corrected.
EPs did not understand the items 7, 8 and 9 that measured the subjective norm construct. In particular, they did not understand whom they should think about when asked what	The concept of "people who are important to me" was difficult to comprehend for certain participants, but we decided to keep the construct as it is, because changing it to "people who

“people who are important to them" would think about their adoption of the behavior being studied. They suggested removing these questions or asking instead about "people who are professionally important to them."	are professionally important to me" would change the meaning of the construct from a more general subjective norm to a specifically professional norm. Moreover, when questioned about whom they thought about when answering this question, all participants stated that they thought about people who were professionally important to them anyhow.
EPs also wondered about the importance of some of the referents. In particular, they could not comprehend that AHPs might influence their decision to use a wiki-based reminder or not and suggested we remove these items.	Considering the importance of these salient beliefs that emerged in our previous qualitative survey of 25 EPs, we decided to leave these items in our final questionnaire even though it was suggested we remove them.
Questions 22 (items concerning facilitators) and 24 (items concerning advantages) in the EP version are long.	Although we did not remove any items at this point, we simplified the wording for a number of items to decrease the length of the question.
Overall, EP and AHP focus group participants understood what a wiki-based reminder was.	No modification was made.
When asked if the participants preferred a paper- or a web-based questionnaire, most participants expressed the preference to use the web-based version because of the embedded video link.	Even though most of our focus group participants preferred the web-based version, we decided to keep the paper-based questionnaire as an option for participants who might prefer paper in our larger sample.
Most AHPs stated that the YouTube video was blocked on their hospital computers.	Quebec's Health System telecommunication network blocks YouTube videos. For participants wanting to use the online survey, we will send a complete copy of the video via a file sharing site and ask the local investigator to install the video on a computer close to the participants’ workplace. We also reformatted our YouTube videos into .wmv and .mpeg files so that they could be watched on a Windows or an iOS system.
**Comments specific to the paper version**	
It was suggested that the html link referred to in the paper-based questionnaire needs to be written out in full for participants to access the video online, or that a better way be found of making the video available to those participants who respond via paper.	We wrote out the YouTube link in full on our paper-based questionnaire so that participants could copy the full url into a web browser. In addition, all participants in our survey will be instructed that a complete version of the video will be made available on a computer in their workplace (emergency department) without them needing to connect to the internet.
