# Supplementary material for: Development and Validation of Questionnaires Exploring Health Care Professionals' Intention to Use Wiki-Based Reminders to Promote Best Practices in Trauma
Source: JMIR Res Protoc. 2014 Oct 3;3(4):e50. doi: 10.2196/resprot.3762 (PMC4213801; doi:10.2196/resprot.3762)
Supplement: Supplementary file 6 [file resprot_v3i3e50_app6.pdf]

Multimedia Appendix 6: **Analysis of internal consistency and temporal stability for each item in the EP and AHP questionnaires**

A) Analysis of internal consistency and temporal stability in the EP questionnaire

1. Perceived behavioral control (Cronbach's alpha: Test = 0.65; Retest = 0.67)

Variable	Correlation with the total TEST	Correlation with the total RETEST	ICC (temporal stability)
Pbc1 (Question1)	0.20	0.26	0.75 (0.50; 0.88)
Pbc2 (Q2)	0.62	0.50	0.58 (0.23; 0.79
Pbc3 (Q4)	0.57	0.59	0.59 (0.27; 0.80)

ICC on the global score (average of the 3 items) = 0.68 (0.39; 0.85)

The question « *Je ne vois pas d'obstacles à utiliser un aide-mémoire basé dans un wiki qui promeut une pratique exemplaire de prise en charge des traumatisés crâniens sévères dans les salles d'urgence du Québec* » affects the internal consistency of the construct.

Cronbach's alpha (2 items): Test = 0.86; Retest = 0.85

ICC on the global score (average of the 2 items) = 0.64 (0.33; 0.82)

Question Q1 (Pbc1) needs to be reformulated.

2. Social norm construct (Cronbach's alpha: Test = 0.79; Retest = 0.78)

Variable	Correlation with the total TEST	Correlation with the total RETEST	ICC (temporal stability)
Sn1 (Q3)	0.51	0.49	0.21 (-0.19; 0.55)

Sn2 (Q5)	0.74	0.64	0.77 (0.54; 0.89)
Sn3 (Q7)	0.69	0.72	0.80 (0.59; 0.91)

ICC on global score (average of the 3 items) = 0.75 (0.51; 0.88)

Item Sn1 does not affect the temporal stability of the construct. Consequently, it does not need to be reformulated.

3. Intention construct (Cronbach's alpha: Test = 0.94; Retest = 0.98)

**Variable**	**Correlation with the total TEST**	**Correlation with the total RETEST**	**ICC (temporal stability)**
Int1 (Q6)	0.78	0.94	0.85 (0.69; 0.93)
Int2 (Q8)	0.93	0.96	0.85 (0.65; 0.94)
Int3 (Q9)	0.93	0.95	0.86 (0.70; 0.94)

ICC on global score (average of the 3 items) = 0.89 (0.75; 0.95)

4. Attitude construct (Cronbach's alpha: Test = 0.74; Retest = 0.72)

**Variable**	**Correlation with the total TEST**	**Correlation with the total RETEST**	**ICC (temporal stability)**

Att1 (Q10-1)	0.75	0.79	0.72 (0.24; 0.89)
Att2 (Q10-2)	0.06	0.02	0.25 (-0.18; 0.59)
Att3 (Q10-3)	0.69	0.80	0.78 (0.57; 0.90)
Att4 (Q10-4)	0.83	0.65	0.65 (0.35; 0.83)

ICC on global score (average of the 4 items) = 0.70 (0.40; 0.86)

The question « *Pour moi, utiliser un aide-mémoire basé dans un wiki qui promeut une pratique exemplaire pour la prise en charge des traumatisés crâniens sévères dans les salles d'urgence du Québec serait …. RELAXANT/STRESSANT* » is not appropriate for the construct.

Cronbach's alpha (3 items): Test = 0.89; Retest = 0.91

ICC on the global score (average of the 3 items) = 0.78 (0.49; 0.91)

Question Q10-2 (Att2) must be removed.

5. <u>Normative belief construct</u> (Cronbach's alpha: Test = 0.83; Retest = 0.87)

Variable	Correlation with the total TEST	Correlation with the total RETEST	ICC (temporal stability)
C_norm1 (Q11-1)	0.75	0.80	0.66 (0.31; 0.84)
C_norm2 (Q11-2)	0.30	0.64	0.59 (0.25; 0.80)
C_norm3 (Q11-3)	0.70	0.67	0.71 (0.43; 0.86)
C_norm4 (Q11-4)	0.41	0.54	0.61 (0.28; 0.81)
C_norm5 (Q11-5)	-0.02	0.20*	0.47 (0.08; 0.74)
C_norm6 (Q11-6)	0.27*	0.34	0.80 (0.58; 0.91)
C_norm7 (Q11-7)	0.83	0.71	0.53 (0.19; 0.76)
C_norm8 (Q11-8)	0.37	0.31	0.72 (0.46; 0.87)
C_norm9 (Q11-9)	0.71*	0.60	0.74 (0.48; 0.88)
C_norm10 (Q11-10)	0.72	0.58	0.54 (0.16; 0.77)
C_norm11 (Q11-11)	0.59	0.76	0.65 (0.33; 0.83)

*These items have missing data: there were 2 missing answers for the test questionnaire (C_norm6 and C_norm9) and 1 missing answer for the retest (C_norm5). The values for these missing elements were imputed by calculating the average value of the other items in this construct.

ICC on the global score (average of the 11 items) = 0.80 (0.59; 0.91)

Overall, this construct is appropriate, however, questions **C_norm5 and C_norm6 must be reformulated because they do not correlate well with the other items.**

6. Control belief construct – facilitator (Cronbach's alpha: Test = 0.97; Retest = 0.85)

**Variable**	**Correlation with the total TEST**	**Correlation with the total RETEST**	**ICC (temporal stability)**
C_cont1 (Q12-a)	0.87	0.52	-0.04 (-0.42; 0.35)
C_cont2 (Q12-b)	0.64	0.16	0.62 (0.31; 0.82)
C_cont3 (Q12-c)	0.87	0.83	0.24 (-0.17, 0.57)
C_cont4 (Q12-d)	0.96	0.63	0.47 (0.11; 0.73)

C_cont5 (Q12-e)	0.77	0.44	0.27 (-0.09; 0.58)
C_cont6 (Q12-f)	0.93	0.67	0.33 (-0.04; 0.63)
C_cont7 (Q12-g)	0.90*	0.28	0.13 (-0.30; 0.51)
C_cont8 (Q12-h)	0.95	0.46	0.18 (-0.19; 0.52)
C_cont9 (Q12-i)	0.87*	0.43*	0.05 (-0.32; 0.43)
C_cont10 (Q12-j)	0.79	0.56	0.63 (0.31; 0.82)
C_cont11 (Q12-k)	0.90	0.67	0.38 (0.01; 0.67)

* These items have missing data: there were 2 missing answers in the test (C_cont7 and C_cont9) and 1 missing answer in the retest (C_cont9). The values for these missing answers were imputed by calculating the average value of the other items in this construct.

ICC on the global score (average for the 11 items) = 0.30 (-0.07; 0.61)

This construct has good internal consistency, but its temporal stability (RETEST) is very problematic. **Consequently, the items of this construct must be reformulated.**

7. <u>Control belief construct – barriers</u> (Cronbach's alpha: Test = 0.58; Retest = 0.67)

Variable	Correlation with the total TEST	Correlation with the total RETEST	ICC (temporal stability)
C_cont12 (Q13-a)	0.40	0.31	0.74 (0.49; 0.88)
C_cont13 (Q12-b)	0.34	0.37	0.82 (0.62; 0.92)
C_cont14 (Q12-c)	0.16	0.28	0.67 (0.38; 0.84)
C_cont15 (Q12-d)	0.38	0.66*	0.72 (0.44; 0.87)
C_cont16 (Q12-e)	0.42	0.58	0.69 (0.40; 0.85)

*This item had one missing answer in the retest questionnaire (C-cont15). The value for this missing answer was imputed by calculating the average value of the other items in this construct.

ICC on the global score (average for the 5 items) = 0.78 (0.56; 0.90)

This construct has good reproducibility, but its internal consistency is problematic. **Items C_cont13 and C_cont14 must be reformulated.**

8. Behavioral belief construct (Cronbach's alpha: Test = 0.94; Retest = 0.86)

**Variable**	**Correlation with the total TEST**	**Correlation with the total RETEST**	**ICC (temporal stability)**
C_comp1 (Q14-a)	0.71	0.53	0.56 (0.23; 0.78)
C_comp2 (Q14-b)	0.88	0.68	0.70 (0.42; 0.86)
C_comp3 (Q14-c)	0.81	0.64	0.36 (-0.05; 0.66)
C_comp4 (Q14-d)	0.90	0.63	0.61 (0.29; 0.81)
C_comp5 (Q14-e)	0.95	0.82	0.30 (-0.12; 0.63)
C_comp6 (Q14-f)	0.79	0.73*	0.73 (0.46; 0.87)
C_comp7 (Q14-g)	0.88	0.78	0.57 (0.24; 0.79)
C_comp8inv	0.43	0.11	0.59 (0.25; 0.80)

(Q14-h)

*This item had one missing answer in the retest questionnaire (C_comp6). The value for this missing answer was imputed by calculating the average value for the other items in this constuct.

ICC on the global score (average for the 8 items) = 0.60 (0.28; 0.81)

Question C_comp8inv could be reformulated, but this is not necessary. It can simply be inverted at the level of the global score.

B) Analysis of internal consistency and temporal stability for the AHP questionnaire (12 nurses, 7 pharmacists and 7 respiratory therapists)

1. Perceived behavioral control (Cronbach's alpha: Test = 0.55; Retest = 0.62)

Variable	Correlation with the total TEST	Correlation with the total RETEST	ICC (temporal stability)
Pbc1	0.53	0.30*	0.45 (0.09; 0.71)
Pbc2	0.32	0.51	0.42 (0.04; 0.69)
Pbc3	0.26	0.49	0.62 (0.31; 0.81)

*This item had one missing answer in the retest questionnaire (Pbc1). The value for this missing answer was imputed by calculating the average value of the other items in this construct.

ICC on the global score (average for the 3 items) = 0.60 (0.30; 0.80)
This construct has a good temporal stability. Its internal consistency is not adequate, but removing this question does not improve the internal consistency.
Consequently, this construct needs to be reviewed.

2. Social norm construct (Cronbach's alpha: Test = 0.47; Retest = 0.82)

Variable	Correlation with the total TEST	Correlation with the total RETEST	ICC (temporal stability)
Sn1	0.08	0.71	0.37 (0.00; 0.66)
Sn2	0.24	0.68	0.47 (0.10; 0.72)
Sn3	0.65	0.84	0.36 (-0.03; 0.65)

ICC on the global score (average for the 3 items) = 0.62 (0.32; 0.81)
Although these items do not seem to have good temporal stability, overall, the construct has good temporal stability. The problem with internal consistency comes from question Sn1. Removing this question produces a Cronbach's alpha for the test and retest of 0.71 and 0.71 respectively. **Consequently, this item needs to be reviewed.**

3. Intention construct (Cronbach's alpha: Test = 0.69; Retest = 0.81)

Variable	Correlation with the total TEST	Correlation with the total RETEST	ICC (temporal stability)
Int1	0.53	0.64	0.32 (-0.06; 0.62)
Int2	0.72	0.59	0.58 (0.25; 0.79)
Int3	0.32	0.77	0.19 (-0.16; 0.51)

ICC on the global score (average for the 3 items) = 0.48 (0.13; 0.73)

This construct is unstable in its internal consistency and temporal stability. If question Int3 is removed, as suggested by the correlation of the test and the ICC, the Cronbach's alpha for the test and retest are respectively 0.83 and 0.69 (inverted with relation to the questionnaires with 3 items). **Consequently, this construct needs to be reviewed.**

4. Attitude construct (Cronbach's alpha: Test = 0.85; Re-Test = 0.87)

Variable	Correlation with the total TEST	Correlation with the total RETEST	ICC (temporal stability)
Att1	0.83	0.73	0.76 (0.54; 0.89)
Att2	0.57	0.61	0.85 (0.69; 0.93)

Att3	0.78	0.71	0.39 (0.00; 0.68)
Att4	0.58	0.85	0.66 (0.38; 0.83)

ICC on the global score (average for the 4 items) = 0.83 (0.65; 0.92)

This construct has good internal consistency and temporal stability.

5. Normative belief construct (Cronbach's alpha: Test = 0.85; Re-Test = 0.90)

**Variable**	**Correlation with the total TEST**	**Correlation with the total RETEST**	**ICC (temporal stability)**
C_norm1	0.63	0.73	0.61 (0.30; 0.80)
C_norm2	0.68	0.79	0.70 (0.43; 0.85)
C_norm3	0.78	0.67	0.36 (-0.03; 0.66)
C_norm4	0.59	0.75	0.58 (0.25; 0.79)
C_norm5	0.39	0.76	0.52 (0.17; 0.75)
C_norm6	0.29	0.26*	0.07 (-0.35; 0.46)
C_norm7	0.45	0.54	0.05 (-0.31; 0.41)
C_norm8	0.30	0.35	0.11 (-0.29; 0.48)
C_norm9	0.78	0.53	0.51 (0.16; 0.75)
C_norm10	0.47	0.67	0.84 (0.68; 0.93)

C_norm11	0.60	0.66*	0.66 (0.36; 0.83)
C_norm12	0.35	0.83	0.62 (0.32; 0.81)

*These items have missing data: there were 2 missing answers in the retest for item C_norm6 and 1 missing answer for the item C_norm11. The values for these missing answers were imputed by calculating the average value of the other items in this construct.

ICC on the global score (average for the 12 items): 0.74 (0.51; 0.88).
This construct has good internal consistency and good temporal stability. **Questions c_norm6, c_norm7 and c_norm8 could be removed without affecting internal consistency or temporal stability.**

6. Control belief construct – facilitator (Cronbach's alpha: Test = 0.72; Retest = 0.94)

**Variable**	**Correlation with the total TEST**	**Correlation with the total RETEST**	**ICC (temporal stability)**
C_cont1	0.29	0.74	0.21 (-0.16; 0.54)
C_cont2	0.48	0.92	-0.03 (-0.43; 0.37)
C_cont3	0.55	0.87*	0.03 (-0.35; 0.41)
C_cont4	0.61	0.93	-0.04 (-0.43; 0.35)
C_cont5	-0.02	0.29	0.18 (-0.23; 0.53)

C_cont6	0.46	0.75	0.24 (-0.16; 0.57)
C_cont7	0.54	0.85	0.20 (-0.22; 0.54)
C_cont8	0.45	0.89	0.09 (-0.33; 0.46)

*This item had one missing answer in the retest questionnaire (C_cont3). The value for this missing answer was imputed by calculating the average value of the other items in this construct.

ICC on the global score (average for the 8 items): -0.05 (-0.43; 0.35)

The temporal stability of this construct is not adequate. Its internal consistency is adequate. After removing question c_cont5, the Cronbach's alpha for the test becomes 0.77 and the retest 0.96. **Question c_cont5 should therefore be removed**.

7. Control belief construct – barrier (Cronbach's alpha: Test = 0.58; Retest = 0.55)

Variable	Correlation with the total TEST	Correlation with the total RETEST	ICC (temporal stability)

C_cont9	0.25	-0.04	0.50 (0.16; 0.73)
C_cont10	0.28	0.47	0.69 (0.43; 0.85)
C_cont11	0.23	0.28	-0.04 (-0.44; 0.35)
C_cont12	0.67	0.57	0.59 (0.25; 0.80)
C_cont13	0.32	0.48	0.58 (0.27; 0.79)
C_cont14	0.19	0.11	0.71 (0.44; 0.86)

ICC on the global score (average for the 6 items): 0.66 (0.37; 0.83).
Even though this construct has a good temporal stability, the internal consistency of this construct is problematic. There are no items that can be removed to improve the Cronbach's alpha. **Consequently, this construct needs to be reviewed.**

8. Behavioral belief construct (Cronbach's alpha: Test = 0.92; Retest = 0.91)

Variable	Correlation with the total TEST	Correlation with the total RETEST	ICC (temporal stability)
C_comp1	0.48	0.58	0.49 (0.14; 0.73)
C_comp2	0.53	0.73	0.67 (0.39; 0.84)
C_comp3	0.84	0.77	0.68 (0.41; 0.84)
C_comp4	0.78	0.73	0.38 (0.02; 0.66)
C_comp5	0.83	0.63	0.67 (0.39; 0.84)
C_comp6	0.82	0.76	0.51 (0.15; 0.75)
C_comp7	0.86	0.76	0.70 (0.43; 0.85)
C_comp8	0.73	0.76	0.83 (0.65; 0.92)

ICC on the global score (average for the 8 items): 0.82 (0.64; 0.91)
The internal consistency and temporal stability are appropriate.

As a rough guide, we followed the ratings suggested by Landis and Koch with agreement levels as follows: 0-0.2 poor, 0.2-0.4 fair, 0.4-0.6 moderate, 0.6-0.8 substantial, and 0.8-1 almost perfect.

Landis JR, Koch GG: The measurement of observer agreement for categorical data. *Biometrics* 1977, 33:159-174.

We considered ICC was adequate if it was superior to 0.40 according to Landis (corresponding to moderate reproducibility).
