# Supplementary material for: Development and Validation of Questionnaires Exploring Health Care Professionals' Intention to Use Wiki-Based Reminders to Promote Best Practices in Trauma
Source: JMIR Res Protoc. 2014 Oct 3;3(4):e50. doi: 10.2196/resprot.3762 (PMC4213801; doi:10.2196/resprot.3762)
Supplement: Supplementary file 7 [file resprot_v3i3e50_app7.pdf]

Multimedia Appendix 7: Changes made to the EP questionnaire after the 2 week test-retest

Before	After
**Je ne vois pas d'obstacle à utiliser** un aide-mémoire basé dans un wiki qui promeut une pratique exemplaire pour la prise en charge des traumatisés crâniens sévères dans les salles d'urgence du Québec.	**Si je le voulais, je suis confiant que je pourrais utiliser un aide-mémoire basé dans un wiki** qui promeut une pratique exemplaire pour la prise en charge des traumatisés crâniens sévères dans les salles d'urgence du Québec.
**Mon utilisation** d'un aide-mémoire basé dans un wiki qui promeut une pratique exemplaire pour la prise en charge des traumatisés crâniens sévères dans les salles d'urgence du Québec **serait approuvée par :**<br><br>Les **personnes qui s'opposent aux soins standardisés approuveraient** de mon centre hospitalier<br><br>Les **personnes moins à l'aise avec l'informatique** de mon centre hospitalier	**Mon utilisation d'un aide-mémoire basé dans un wiki** qui promeut une pratique exemplaire pour la prise en charge des traumatisés crâniens sévères dans les salles d'urgence du Québec **serait désapprouvée par :**<br><br>Les **personnes qui s'opposent aux soins standardisés** de mon centre hospitalier<br><br>Les **personnes moins à l'aise avec l'informatique** de mon centre hospitalier
**Il serait plus facile pour moi** d'utiliser un aide-mémoire basé dans un wiki qui promeut la meilleure pratique de prise en charge des traumatisés crâniens sévères au département d'urgence au Québec...<br><br>a) **s'il** était simple à utiliser (e.g., la navigation et la recherche d'information)<br><br>c) **si** la qualité scientifique de l'information était validée<br><br>f) **s'il** était compatible avec mon processus de travail	**Je me sentirais capable d'utiliser un aide-mémoire basé dans un wiki** qui promeut la meilleure pratique de prise en charge des traumatisés crâniens sévères au département d'urgence au Québec,...<br><br>a) **s'il** était simple à utiliser.<br><br>**c) si** l'information était validée par un comité de pairs.<br><br>f) **s'il** était bien intégré dans les activités de mon travail.

h) **s'il** était facile de l'adapter à la réalité de mon milieu de travail.<br><br>i) **si** je pouvais l'expérimenter avant de l'utiliser.	SUPPRIMER<br><br>SUPPRIMER
Je me **sentirais capable** d'utiliser un aide-mémoire basé dans un wiki qui promeut la meilleure pratique de prise en charge des traumatisés crâniens sévères au département d'urgence au Québec...<br><br>**b)** même si **j'avais des contraintes de temps**<br><br>c) même si **l'information changeait fréquemment**	b) **même si** j'avais peu de temps.<br><br>c) **même si** l'information changeait souvent
**Si j'utilisais** un aide-mémoire basé dans un wiki qui promeut la meilleure pratique de prise en charge des traumatisés crâniens sévères au département d'urgence au Québec, cela...<br><br>h) **augmenterait** mon niveau de stress	h) **diminuerait** mon niveau de stress
