# Supplementary material for: Development and Validation of Questionnaires Exploring Health Care Professionals' Intention to Use Wiki-Based Reminders to Promote Best Practices in Trauma
Source: JMIR Res Protoc. 2014 Oct 3;3(4):e50. doi: 10.2196/resprot.3762 (PMC4213801; doi:10.2196/resprot.3762)
Supplement: Supplementary file 8 [file resprot_v3i3e50_app8.pdf]

Multimedia Appendix 8. Changes made to the AHP questionnaire after the 2 week test-retest

BEFORE	AFTER
**Les questions PBC n'avais pas assez de cohérence interne Cronbach alpha a 0.55, donc étant donné que la question avait été changé chez les MDs, nous avons aussi profité du manque de cohérence interne pour changer la version Autres**	**Nouvelle Q2: Si je le voulais, je suis confiant que je pourrais utiliser un aide-mémoire basé dans un wiki** qui promeut une pratique exemplaire pour la prise en charge des traumatisés crâniens sévères dans les salles d'urgence du Québec.
**Q3 (ns1) a été reformulé tel que suggéré par ST dans son analyse: La vielle question était: La plupart des personnes qui sont importantes pour moi me recommanderaient d'utiliser un aide-mémoire basé dans un wiki** qui promeut une pratique exemplaire pour la prise en charge des traumatisés crâniens sévères dans les salles d'urgence du Québec.	**Q3 L'item 1 (NS1) a été reformulé pour ressembler au questionnaire MD: Je ressens une pression sociale à utiliser un aide-mémoire basé dans un wiki** qui promeut une pratique exemplaire pour la prise en charge des traumatisés crâniens sévères dans les salles d'urgence du Québec.
**Q6-8-9 traitant de l'INT 1-2-3 n'étaient ni stables dans le temps, ni fiables. La Q9 (int 3) a été modifié pour préciser encore plus la notion de temps.**	**Q9 a été changé comme suit: Dans le futur, j'utiliserai un aide-mémoire basé dans un wiki** qui promeut une pratique exemplaire pour la prise en charge des traumatisés crâniens sévères dans les salles d'urgence du Québec.
**Q11** Les items c_norm6, c_norm7 et c_norm8 peuvent être enlevés sans affecter la consistance et la stabilité temporelle. Nous avons donc décidé d'enlever la c_norm6 et 8. Étant donné que ces trois croyances étaient des référents négatifs, nous avons donc décidé tout de même de laisser la croyance normative: «les personnes moins à l'aise avec l'informatique».	**Q11. les items 6 et 8 ont été enlevés: ces items faisaient référence aux personnes ne voulants pas suivre les soins standardisés et les personnes réfractaires au changement.**
**Q13 les questions par rapport aux barrières n'étaient pas fiables, le nombre total d'item a été réduit en enlevant les items moins intéressants selon leur rang dans les croyances saillantes**	**Q13 L'item sur le coût des ordinateurs a été enlevé**
**Q12e:** Le facilitateur «s'il y avait de la publicité sur l'aide-mémoire	**Q12:** La question 12 n'a maintenant que 7 items, en enlevant l'item

basé dans un wiki» a été enlevé car la stabilité dans le temps n'était pas bonne pour tout ce construit. Donc en diminuant la lassitude des participants on veut augmenter l'ICC (stabilité dans le temps). Il y avait 8 items initialement.	Q12e.
