# Supplementary material for: Development and Validation of Questionnaires Exploring Health Care Professionals' Intention to Use Wiki-Based Reminders to Promote Best Practices in Trauma
Source: JMIR Res Protoc. 2014 Oct 3;3(4):e50. doi: 10.2196/resprot.3762 (PMC4213801; doi:10.2196/resprot.3762)
Supplement: Supplementary file 9 [file resprot_v3i3e50_app9.pdf]

ÉTUDE SUR L'UTILISATION DU WIKI

QUESTIONNAIRE

PROJET WIKI (B)

Date : ________________
jj/mm/aaaa

1. Ce questionnaire porte sur votre utilisation d'un aide-mémoire basé dans un wiki qui promeut une pratique exemplaire pour la prise en charge des traumatisés crâniens sévères dans les salles d'urgence du Québec.

2. Bien que certaines questions puissent vous sembler répétitives, il est très important de répondre à toutes les questions.

3. Pour répondre aux questions, vous devez inscrire votre réponse à l'endroit reflétant le mieux votre opinion ou votre situation.

4. Notez qu'il n'y a ni bonne ni mauvaise réponse.

5. Vos réponses demeureront confidentielles.

6. Le temps requis pour répondre au questionnaire est d'environ 10 minutes.

La participation à ce sondage est volontaire et vos données resteront confidentielles. Le simple fait d'acheminer le questionnaire rempli sera considéré comme l'expression de votre consentement à participer au projet.

Pour tout commentaire ou question concernant ce projet de recherche, veuillez communiquer avec Susie Gagnon, professionnelle de recherche au 418-835-7121 poste 6267.

Consentement:

Je comprends que les données de ce questionnaire sont anonymes. Je comprends que les données seront traitées de façon confidentielle et, qu'en aucun cas, elles ne seront associées à mon établissement lors de la diffusion des résultats. Après avoir pris connaissance des informations ci-dessus, j'accepte librement de participer à cette étude et j'ai bien compris que je peux arrêter de répondre au questionnaire n'importe quand, sans préjudice.

☐ Oui

☐ Non

Avant de débuter, il faut regarder la vidéo suivante :

Infirmier(ère), cliquez ici

Inhalothérapeute, cliquez ici

Pharmacien(ne), cliquez ici

Voici la définition de l'utilisation d'un wiki :

• Un wiki est un site Web programmé pour permettre l'édition par toute personne y ayant accès. Il est donc un outil de travail collaboratif et contient par exemple des protocoles de soins et des algorithmes de décision. Plus concrètement, dans le monde de la santé, un wiki pourrait permettre aux médecins et autres professionnels de partager, mettre à jour et éditer des aide-mémoire selon les dernières données probantes. Une fois implanté dans un centre hospitalier, il permettrait à tous les professionnels de la santé y ayant accès de le consulter directement sur leurs lieux de travail.

• Par exemple, dans le cas d'un traumatisme crânien sévère, l'aide-mémoire basé dans un wiki pourra être trouvé sur le web à partir d'un ordinateur et ainsi l'équipe pourra l'utiliser pour intervenir.

Ce questionnaire porte sur votre opinion concernant l'utilisation potentielle d'un aide-mémoire basé dans un wiki faisant la promotion des pratiques exemplaires de prise en charge des traumatisés crâniens sévères dans les salles d'urgence du Québec.

1. **Je ne vois pas d'obstacle à utiliser un aide-mémoire basé dans un wiki** qui promeut une pratique exemplaire pour la prise en charge des traumatisés crâniens sévères dans les salles d'urgence du Québec.

1	2	3	4	5	6	7
Très en désaccord						Très en accord

2. **Si je le voulais, je suis confiant que je pourrais utiliser un aide-mémoire basé dans un wiki** qui promeut une pratique exemplaire pour la prise en charge des traumatisés crâniens sévères dans les salles d'urgence du Québec.

1	2	3	4	5	6	7
Très en désaccord						Très en accord

3. **Je ressens une pression sociale à utiliser un aide-mémoire basé dans un wiki** qui promeut une pratique exemplaire pour la prise en charge des traumatisés crâniens sévères dans les salles d'urgence du Québec.

1	2	3	4	5	6	7
Très en désaccord						Très en accord

4. **Pour moi, utiliser un aide-mémoire basé dans un wiki** qui promeut la meilleure pratique de prise en charge des traumatisés crâniens sévères au département d'urgence au Québec serait...

1	2	3	4	5	6	7
Très difficile						Très facile

5. **Si j’utilisais un aide-mémoire basé dans un wiki** qui promeut une pratique exemplaire pour la prise en charge des traumatisés crâniens sévères dans les salles d’urgence du Québec, la plupart des personnes qui sont importantes pour moi seraient...

1	2	3	4	5	6	7
**Très en désaccord**						**Très en accord**

6. **J’ai l’intention d’utiliser un aide-mémoire basé dans un wiki** qui promeut la meilleure pratique de prise en charge des traumatisés crâniens sévères dans les départements d’urgence du Québec.

1	2	3	4	5	6	7
**Très en désaccord**						**Très en accord**

7. **Les personnes les plus importantes pour moi pensent que je devrais utiliser un aide-mémoire basé dans un wiki** qui promeut une pratique exemplaire pour la prise en charge des traumatisés crâniens sévères dans les salles d’urgence du Québec.

1	2	3	4	5	6	7
**Très en désaccord**						**Très en accord**

8. **J’évalue mes chances d’utiliser un aide-mémoire basé dans un wiki** qui promeut une pratique exemplaire pour la prise en charge des traumatisés crâniens sévères dans les salles d’urgence du Québec comme étant :

1	2	3	4	5	6	7
**Très faibles**						**Très grandes**

9. **Dans le futur, j'utiliserai un aide-mémoire basé dans un wiki** qui promeut une pratique exemplaire pour la prise en charge des traumatisés crâniens sévères dans les salles d’urgence du Québec.

1	2	3	4	5	6	7
**Très improbable**						**Très probable**

10. **Pour moi, utiliser un aide-mémoire basé dans un wiki** qui promeut une pratique exemplaire pour la prise en charge des traumatisés crâniens sévères dans les salles d’urgence du Québec serait...
[Cochez la case appropriée pour ***chacun*** ***des trois énoncés suivants****]*

1	2	3	4	5	6	7

**Très désagréable**	**Très agréable**

1	2	3	4	5	6	7
**Très stressant**						**Très relaxant**

1	2	3	4	5	6	7
**Très inutile**						**Très utile**

1	2	3	4	5	6	7
**Très insatisfaisant**						**Très satisfaisant**

Afin de répondre au groupe de questions suivant, s.v.p. veuillez vous référer à l'échelle de réponse suivante :

1	2	3	4	5	6	7
**Très en désaccord**						**Très en accord**

11. **Mon utilisation d'un aide-mémoire basé dans un wiki** qui promeut une pratique exemplaire pour la prise en charge des traumatisés crâniens sévères dans les salles d'urgence du Québec **serait approuvée par :**

Les **médecins** de mon centre hospitalier

1	2	3	4	5	6	7

Les **inhalothérapeutes** de mon centre hospitalier

1	2	3	4	5	6	7

Le **personnel infirmier** de mon centre hospitalier

1	2	3	4	5	6	7

Le **personnel hospitalier des centres éloignés, moins exposés aux traumatisés crâniens sévères**

1	2	3	4	5	6	7

La **génération plus jeune d'employés** de mon centre hospitalier

1	2	3	4	5	6	7

Les **personnes moins à l'aise avec l'informatique** de mon centre hospitalier

1	2	3	4	5	6	7

L'**équipe de traumatologie** de mon centre hospitalier

1	2	3	4	5	6	7

Les **personnes responsables de la qualité des soins** de mon centre hospitalier

1	2	3	4	5	6	7

L'**administration** de mon centre hospitalier

1	2	3	4	5	6	7

Mes **patients**

1	2	3	4	5	6	7

12. **Il serait plus facile pour moi d'utiliser un aide-mémoire basé dans un wiki** qui promeut une pratique exemplaire pour la prise en charge des traumatisés crâniens sévères dans les salles d'urgence du Québec... *[Cochez la case appropriée pour* ***chacun des sept énoncés suivants****]*

	Très en désaccord	Assez en désaccord	Légèrement en désaccord	Ni en désaccord ni en accord	Légèrement en accord	Assez en accord	Très en accord
a) si l'ordinateur était accessible au chevet du patient.							
b) si la qualité scientifique de l'information était validée.							
c) si je pouvais l'expérimenter avant de l'utiliser							
d) s'il était simple à utiliser (e.g. la navigation, la recherche d'information).							
e) si le site était sécurisé.							
f) s'il y avait un poste de travail pour chaque profession.							
g) s'il avait une conception visuelle de qualité.							

13. **Je me sentirais capable d'utiliser un aide-mémoire basé dans un wiki** qui promeut une pratique exemplaire pour la prise en charge des traumatisés crâniens sévères dans les salles d'urgence du Québec…
[Cochez la case appropriée pour ***chacun*** ***des cinq énoncés suivants****]*

	**Très en désaccord**	**Assez en désaccord**	**Légèrement en désaccord**	**Ni en désaccord ni en accord**	**Légèrement en accord**	**Assez en accord**	**Très en accord**
a) **même si** j'ignorais qui détient la responsabilité légale.							
b) **même si** j'avais des contraintes de temps.							
c) **même si** le système informatique n'était pas toujours fiable.							
d) **même si** ce n'est pas toute l'équipe qui est à jour concernant la dernière version de l'aide-mémoire.							
e) **même si** son utilisation était obligatoire.							

14. **Si j'utilisais** un aide-mémoire basé dans un wiki qui promeut une pratique exemplaire pour la prise en charge des traumatisés crâniens sévères dans les salles d'urgence du Québec, cela...
*[Cochez la case appropriée pour **chacun** **des huit énoncés suivants**]*

	**Très en désaccord**	**Assez en désaccord**	**Légèrement en désaccord**	**Ni en désaccord ni en accord**	**Légèrement en accord**	**Assez en accord**	**Très en accord**
a) **augmenterait la rapidité d'accès aux aide-mémoire.**							
b) **améliorerait la qualité des soins que je donne.**							
c) me permettrait d'**avoir des aide-mémoire mis à jour régulièrement.**							
d) **uniformiserait les pratiques.**							
e) **favoriserait le travail d'équipe.**							
f) **centraliserait l'information et les protocoles.**							
g) me donnerait **accès aux données probantes.**							
h) servirait d'**outil pour l'enseignement.**							

Données sociodémographiques

15. Quel âge avez-vous? ______ ans

16. Quel est votre sexe?

☐ Femme
☐ Homme

17. Quelle est votre profession?

☐ Infirmier(ère)
☐ Inhalothérapeute
☐ Pharmacien(ne)
☐ Autre________________________________

18. Dans quel centre hospitalier travaillez-vous? ________________________

19. Depuis combien d'années exercez-vous votre métier (après les stages)?___________

20. a) Y a-t-il un ordinateur avec accès à Internet dans votre salle d'urgence?
☐ Oui
☐ Non

21. Utilisez-vous actuellement un wiki pour un usage professionnel (e.g., Wikipédia)?
☐ Oui → Lequel et à quelle fréquence? ______________________________
☐ Non

22. Utilisez-vous actuellement un wiki pour un usage personnel (e.g., Wikipédia)?
☐ Oui → Lequel et à quelle fréquence? ______________________________
☐ Non

23. Avez-vous édité, par le passé, un wiki?
☐ Oui → Lequel? ______________________________
☐ Non

24. Êtes-vous membre d'un comité de traumatologie (local ou régional)?
☐ Oui
☐ Non

Le questionnaire est terminé!

S'il vous plaît, assurez-vous d'avoir répondu à toutes les questions.

MERCI DE VOTRE PRÉCIEUSE COLLABORATION

Si vous avez des commentaires ou suggestions concernant ce questionnaire ou cette recherche, vous pouvez les inscrire ci-dessous.

____
____
____
____
____
