# Supplementary material for: Development and Validation of Questionnaires Exploring Health Care Professionals' Intention to Use Wiki-Based Reminders to Promote Best Practices in Trauma
Source: JMIR Res Protoc. 2014 Oct 3;3(4):e50. doi: 10.2196/resprot.3762 (PMC4213801; doi:10.2196/resprot.3762)
Supplement: Supplementary file 11 [file resprot_v3i3e50_app11.pdf]

STUDY ON WIKI USE

QUESTIONNAIRE

WIKI PROJECT (B)

Date : ________________
dd/mm/yyyy

1. This questionnaire is about the use of wiki-based reminders that promote best practice in the management of severe cranial trauma in emergency rooms in the province of Quebec.
2. Although some of the questions may seem repetitive, it is very important that you answer them all anyway.
3. Your choice of answers should best reflect your opinion or your situation.
4. There are no right and wrong answers.
5. Your answers will remain confidential.
6. The questionnaire should take about 10 minutes.

Your participation in this survey is voluntary and your data will remain confidential. Returning the completed questionnaire will be considered an expression of your consent to participate in the project. For any comments or questions about this research project please contact Susie Gagnon, Research Professional, at 418-835-7121 ext. 6267.

Consent:

I understand that the data collected by this questionnaire will be anonymous. I understand that it will be treated with confidentiality and in no circumstances will be associated with my institution when the results are made public. I have noted the above information and I freely consent to participate in this study. I understand that I may stop answering the questionnaire at any moment without prejudice.

☐ Yes

☐ No

Before you begin, please watch the following video depending on your profession:

Nurses' video

Respiratory Therapists' video

Pharmacists' video

Definition of a wiki

- A wiki is a website programmed to allow anyone who has access to it to edit (change) its content. It is thus a collaborative work tool, and could contain treatment protocols, for example, or decision algorithms. In the healthcare context, a wiki could enable doctors and

other professionals to share, update and edit reminders based on the most recent evidence. Healthcare professionals who had access to a wiki in their hospital could consult it directly in their workplace.

• For example, in the case of a severe cranial trauma, a wiki-based reminder could be brought up online on a computer and the team could use the information in their response.

This questionnaire seeks your opinion on the possible use of a wiki-based reminder that promotes best practices in managing severe cranial trauma in emergency rooms in the province of Quebec.

1. **I see no obstacles to using a wiki-based reminder** that promotes best practice for managing severe cranial trauma in emergency rooms in the province of Quebec.

1	2	3	4	5	6	7
**Strongly disagree**						**Strongly agree**

2. **If I wanted to, I feel confident that I could use a wiki-based reminder** that promotes best practice for managing severe cranial trauma in emergency rooms in the province of Quebec.

1	2	3	4	5	6	7
**Strongly disagree**						**Strongly agree**

3. **I feel social pressure to use a wiki-based reminder** that promotes best practice for managing severe cranial trauma in emergency rooms in the province of Quebec.

1	2	3	4	5	6	7
**Strongly disagree**						**Strongly agree**

4. **For me to use a wiki-based reminder** that promotes best practice for managing severe cranial trauma in emergency rooms in the province of Quebec would be:

1	2	3	4	5	6	7

Very difficult **Very easy**

5. **If I used a wiki-based reminder** that promotes best practice for managing severe cranial trauma in emergency rooms in the province of Quebec, most of the people who are important to me would:

1 2 3 4 5 6 7

Strongly disagree **Strongly agree**

6. **I intend to use a wiki-based reminder** that promotes best practice for managing severe cranial trauma in emergency rooms in the province of Quebec.

1 2 3 4 5 6 7

Strongly disagree **Strongly agree**

7. **The people who are most important to me think I should use a wiki-based reminder** that promotes best practice for managing severe cranial trauma in emergency rooms in the province of Quebec.

1 2 3 4 5 6 7

Strongly disagree **Strongly agree**

8. **I estimate the odds of my using a wiki-based reminder** that promotes best practice for managing severe cranial trauma in emergency rooms in the province of Quebec to be:

1 2 3 4 5 6 7

Very low **Very high**

9. **In the future I will use a wiki-based reminder** that promotes best practice for managing severe cranial trauma in emergency rooms in the province of Quebec.

1 2 3 4 5 6 7

Very unlikely **Very likely**

10. **For me, using a wiki-based reminder** that promotes best practice for managing severe cranial trauma in emergency rooms in the province of Quebec would be:

*[tick **one box** in **each** of these 4 scales]*

1	2	3	4	5	6	7
**Very unpleasant**						**Very pleasant**

1	2	3	4	5	6	7
**Very stressful**						**Very relaxing**

1	2	3	4	5	6	7
**Totally useless**						**Very useful**

1	2	3	4	5	6	7
**Very unsatisfying**						**Very satisfying**

Please use this scale to answer the question that follows.

1	2	3	4	5	6	7
**Strongly disagree**						**Strongly agree**

11. The following people would **approve** of **my use of a wiki-based reminder** that promotes best practice for managing severe cranial trauma in emergency rooms in the province of Quebec**:**

The **doctors** at my hospital

1 2 3 4 5 6 7

The **respiratory therapists** at my hospital

1 2 3 4 5 6 7

The **nurses** at my hospital

1 2 3 4 5 6 7

The hospital staff in regional/remote hospitals who are less exposed to severe cranial trauma

1 2 3 4 5 6 7

The **younger generation of staff** at my hospital

1 2 3 4 5 6 7

People at my hospital **who are not used to computers**

1 2 3 4 5 6 7

The **trauma team** at my hospital

1 2 3 4 5 6 7

The **people responsible for quality of care** at my hospital

1 2 3 4 5 6 7

The **administration** at my hospital

1 2 3 4 5 6 7

My **patients**

1 2 3 4 5 6 7

12. **It would be easier for me to use a wiki-based reminder** that promotes best practice for managing severe cranial trauma in emergency rooms in the province of Quebec:
[*Tick* ***one box*** *for* ***each*** *of the 7 statements below*]

	Strongly disagree	More or less disagree	Slightly disagree	Neither agree or disagree	Slightly agree	More or less agree	Strongly agree
a) if the computer was available at the patient's bedside.							
b) if the scientific quality had been validated.							
c) if I could try it out before using it.							
d) if it was simple to use (e.g. navigation, searching for information)							
e) if it was a secure site.							
f) if there was a workstation for each profession.							
g) if it was well designed.							

13. **I would feel capable of using a wiki-based reminder** that promotes best practice for managing severe cranial trauma in emergency rooms in the province of Quebec:

[*Tick **one box** for **each** of the 5 statements below*]

	Strongly disagree	More or less disagree	Slightly disagree	Neither agree or disagree	Slightly agree	More or less agree	Strongly agree
a) **even if** I didn't know who was legally responsible.							
b) **even if** I had time constraints.							
c) **even if** the computer system wasn't always reliable.							
d) **even if** not everyone in the team was up to speed with the latest version of the reminder							
e) **even if** its use was obligatory.							

14. **If I used** a wiki-based reminder that promotes best practice for managing severe cranial trauma in emergency rooms in the province of Quebec, it would:
[*Tick **one box** for **each** of the 8 statements below*]

	Strongly disagree	More or less disagree	Slightly disagree	Neither agree or disagree	Slightly agree	More or less agree	Strongly agree
a) **speed up access to reminders.**							
b) **improve the quality of care that I provide.**							
c) give me **reminders that were regularly updated.**							
d) **standardize practices.**							
e) **promote team work.**							
f) **centralize information and protocols.**							
g) give me **access to evidence**							
h) be useful as a **teaching tool.**							

Sociodemographic information

15. How old are you? ______

16. What sex are you?
☐ Woman
☐ Man

17. What is your profession?
☐ Nurse
☐ Respiratory Therapist
☐ Pharmacist
☐ Other________________________________

18. What hospital do you work in? ________________________

19. How many years have you been practising your profession (after interning)?

20. a) Is there a computer with internet access in your emergency room?

☐ Yes

☐ No

21. Do you currently use a wiki in your professional life (e.g. Wikipedia)

☐ Yes → Which one and how often? __________________________

☐ No

22. Do you currently use a wiki in your personal life?

☐ Yes → Which one and how often? _________________________

☐ No

23. Have you ever edited a wiki (made changes to the website)?

☐ Yes → Which one? ____________________________

☐ No

24. Are you a member of a traumatology committee (local or regional)?

☐ Yes

☐ No

The questionnaire is over!

Please make sure you have answered all the questions.

THANK YOU FOR YOUR COLLABORATION

If you have any comments or suggestions regarding this questionnaire or this study please write them here:
