# Supplementary material for: Development and Validation of Questionnaires Exploring Health Care Professionals' Intention to Use Wiki-Based Reminders to Promote Best Practices in Trauma
Source: JMIR Res Protoc. 2014 Oct 3;3(4):e50. doi: 10.2196/resprot.3762 (PMC4213801; doi:10.2196/resprot.3762)
Supplement: Supplementary file 12 [file resprot_v3i3e50_app12.pdf]

Centre de recherche du
Centre hospitalier affilié universitaire
Hôtel-Dieu de Lévis (CHAU)

# STUDY ON WIKI USE

## *PHYSICIAN QUESTIONNAIRE*

***WIKI PROJECT (A)***

**Date:** ________________
dd/mm/yyyy

**1.** This questionnaire is about the use of wiki-based reminders that promote best practice in the management of severe cranial trauma in emergency rooms in the province of Quebec.
**2.** Although some of the questions may seem repetitive, it is very important that you answer them all anyway.
**3.** Your choice of answers should best reflect your opinion or your situation.
**4.** There are no right and wrong answers.
**5.** Your answers will remain confidential.
**6.** The questionnaire should take about 10 minutes.

**Your participation in this survey is voluntary and your data will remain confidential. Returning the completed questionnaire will be considered an expression of your consent to participate in the project.**

**For any comments or questions about this research project please contact Susie Gagnon, Research Professional, at 418-835-7121 ext. 6267.**

**Consent:**

I understand that the data collected by this questionnaire will be anonymous. I understand that it will be treated with confidentiality and in no circumstances will be associated with my institution when the results are made public. I have noted the above information and I freely consent to participate in this study. I understand that I may stop answering the questionnaire at any moment without prejudice.

- [ ] Yes
- [ ] No

**Before you begin, please watch this video.**

## Definition of a wiki:

- A wiki is a website programmed to allow anyone who has access to it to edit (change) its content. It is thus a collaborative work tool, and could contain treatment protocols, for example, or decision algorithms. In the healthcare context, a wiki could enable doctors and other professionals to share, update and edit reminders based on the most recent evidence. Healthcare professionals who had access to a wiki in their hospital could consult it directly in their workplace.
- For example, in the case of a severe cranial trauma, a wiki-based reminder could be brought up online on a computer and the team could use the information in their response.

**This questionnaire seeks your opinion on the possible use of a wiki-based reminder that promotes best practices in managing severe cranial trauma in emergency rooms in the province of Quebec.**

1. **I would feel capable of using a wiki-based reminder** that promotes best practice for managing severe cranial trauma in emergency rooms in the province of Quebec.

| 1 | 2 | 3 | 4 | 5 | 6 | 7 |
|---|---|---|---|---|---|---|
| **Strongly disagree** | | | | | | **Strongly agree** |

2. **If I wanted to, I feel confident that I could use a wiki-based reminder** that promotes best practice for managing severe cranial trauma in emergency rooms in the province of Quebec.

| 1 | 2 | 3 | 4 | 5 | 6 | 7 |
|---|---|---|---|---|---|---|
| **Strongly disagree** | | | | | | **Strongly agree** |

3. **I feel social pressure to use a wiki-based reminder** that promotes best practice for managing severe cranial trauma in emergency rooms in the province of Quebec.

| 1 | 2 | 3 | 4 | 5 | 6 | 7 |
|---|---|---|---|---|---|---|
| **Strongly disagree** | | | | | | **Strongly agree** |

4. **For me to use a wiki-based reminder** that promotes best practice for managing severe cranial trauma in emergency rooms in the province of Quebec would be:

| 1 | 2 | 3 | 4 | 5 | 6 | 7 |
|---|---|---|---|---|---|---|
| **Very difficult** | | | | | | **Very easy** |

5. **I am expected to use a wiki-based reminder** that promotes best practice for managing severe cranial trauma in emergency rooms in the province of Quebec.

| 1 | 2 | 3 | 4 | 5 | 6 | 7 |
|---|---|---|---|---|---|---|
| **Strongly disagree** | | | | | | **Strongly agree** |

6. **I intend to use a wiki-based reminder** that promotes best practice for managing severe cranial trauma in emergency rooms in the province of Quebec.

| 1 | 2 | 3 | 4 | 5 | 6 | 7 |
|---|---|---|---|---|---|---|
| **Strongly disagree** | | | | | | **Strongly agree** |

7. **The people who are most important to me think I should use a wiki-based reminder** that promotes best practice for managing severe cranial trauma in emergency rooms in the province of Quebec.

| 1 | 2 | 3 | 4 | 5 | 6 | 7 |
|---|---|---|---|---|---|---|
| **Strongly disagree** | | | | | | **Strongly agree** |

8. **I estimate the odds of my using a wiki-based reminder** that promotes best practice for managing severe cranial trauma in emergency rooms in the province of Quebec to be:

| 1 | 2 | 3 | 4 | 5 | 6 | 7 |
|---|---|---|---|---|---|---|
| **Very low** | | | | | | **Very high** |

9. **I will use a wiki-based reminder** that promotes best practice for managing severe cranial trauma in emergency rooms in the province of Quebec.

| 1 | 2 | 3 | 4 | 5 | 6 | 7 |
|---|---|---|---|---|---|---|
| **Very unlikely** | | | | | | **Very likely** |

10. **For me, using a wiki-based reminder** that promotes best practice for managing severe cranial trauma in emergency rooms in the province of Quebec would be:

**(***tick* ***one box*** *in* ***each*** *of these three scales***)**

| 1 | 2 | 3 | 4 | 5 | 6 | 7 |
|---|---|---|---|---|---|---|
| **Very unpleasant** | | | | | | **Very pleasant** |

| 1 | 2 | 3 | 4 | 5 | 6 | 7 |
|---|---|---|---|---|---|---|
| **Totally useless** | | | | | | **Very useful** |

| 1 | 2 | 3 | 4 | 5 | 6 | 7 |
|---|---|---|---|---|---|---|
| **Very unsatisfying** | | | | | | **Very satisfying** |

**Please use this scale to answer the question that follows.**

| 1 | 2 | 3 | 4 | 5 | 6 | 7 |
|---|---|---|---|---|---|---|
| **Strongly disagree** | | | | | | **Strongly agree** |

11. The following people would **approve** of **my use of a wiki-based reminder** that promotes best practice for managing severe cranial trauma in emergency rooms in the province of Quebec:

The **nurses** at my hospital

**1 2 3 4 5 6 7**

The **doctors** at my hospital

**1 2 3 4 5 6 7**

**The hospital staff in regional/remote hospitals who are less exposed to severe cranial trauma**

**1 2 3 4 5 6 7**

The **younger generation of staff** at my hospital

**1 2 3 4 5 6 7**

The **respiratory therapists** at my hospital

**1 2 3 4 5 6 7**

The **trauma team** at my hospital

**1 2 3 4 5 6 7**

The **administration** at my hospital

**1 2 3 4 5 6 7**

My **patients**

**1 2 3 4 5 6 7**

The **specialists (intensive care, surgeons)** at my hospital

**1 2 3 4 5 6 7**

**Please use this scale to answer the question that follows.**

**1 2 3 4 5 6 7**

**Strongly disagree** **Strongly agree**

12. The following people would **disapprove** of **my use of a wiki-based reminder** that promotes best practice for managing severe cranial trauma in emergency rooms in the province of Quebec would be disapproved of by

People who are **opposed to the standardized care** offered at my hospital

**1 2 3 4 5 6 7**

People at my hospital **who are not used to computers**

**1 2 3 4 5 6 7**

13. **I would feel capable of using a wiki-based reminder** that promotes best practice for managing severe cranial trauma in emergency rooms in the province of Quebec:
[*Tick* ***one box*** *for* ***each*** *of the 9 statements below*]

| | Strongly disagree | More or less disagree | Slightly disagree | Neither agree or disagree | Slightly agree | More or less agree | Strongly agree |
|---|---|---|---|---|---|---|---|
| a) **if** it was simple to use. | | | | | | | |
| b) **if** a computer was available at the patient's bedside | | | | | | | |
| c) **if** the information had been validated by a peer review committee. | | | | | | | |
| d) **if** access to the reminder was quick. | | | | | | | |
| e) **if** the institutional did not control access (e.g. passwords, blocked sites). | | | | | | | |
| f) **if** it was properly integrated into my work activities. | | | | | | | |
| g) **if** it was accessible via my mobile device (e.g. iPod, iPhone, iPad, Blackberry). | | | | | | | |
| j) **if** my hospital had internet access. | | | | | | | |
| k) **if** the site was well designed. | | | | | | | |

14. **I would feel capable of using a wiki-based reminder** that promotes best practice for managing severe cranial trauma in emergency rooms in the province of Quebec:
[*Tick* ***one box*** *for* ***each*** *of the 5 statements below*]

| | **Strongly disagree** | **More or less disagree** | **Slightly disagree** | **Neither agree or disagree** | **Slightly agree** | **More or less agree** | **Strongly agree** |
|---|---|---|---|---|---|---|---|
| a) **even if** the information wasn't regularly updated. | | | | | | | |
| b) **even if** I had very little time. | | | | | | | |
| c) **even if** the information changed often. | | | | | | | |
| d) **even if** the authors were not identified. | | | | | | | |
| e) **even if** I didn't know who was legally responsible. | | | | | | | |

15. **If I used a wiki-based reminder** that promotes best practice for managing severe cranial trauma in emergency rooms in the province of Quebec, it would:
[*Tick **one box** for **each** of the 8 statements below*]

| | Strongly disagree | More or less disagree | Slightly disagree | Neither agree or disagree | Slightly agree | More or less agree | Strongly agree |
|---|---|---|---|---|---|---|---|
| a) enable me to refresh my memory. | | | | | | | |
| b) give me access to evidence-based information. | | | | | | | |
| c) enable sharing of information with other hospitals. | | | | | | | |
| d) standardize practices. | | | | | | | |
| e) centralize information and protocols. | | | | | | | |
| f) reduce intervention errors. | | | | | | | |
| g) give me access to expert opinion. | | | | | | | |
| h) reduce my stress level. | | | | | | | |

**Sociodemographic information**

16. How old are you? ______

17. What sex are you?
F ☐ M ☐

18. Do you have a certificate in emergency medicine?
Yes ☐→ ☐ College of Family Physicians of Canada
☐ Royal College of Physicians and Surgeons of _______

No ☐→ My specialty is ____________________________

19. What hospital do you work in? ________________________

20. How many years have you worked in emergency (since your residency)? _____

21. Is there a computer with internet access in your emergency room?
Yes ☐ No ☐

22. Do you currently use a wiki in your professional life (e.g. Wikipedia)
☐ Yes → Which one? _______________How often?_____________
☐ No

23. Do you currently use a wiki in your personal life?
☐ Yes → Which one? _______________How often? _____________
☐ No

24. Have you ever edited a wiki (made changes to the website)?
☐ Yes → Which one? _______________ How often? _____________
☐ No

25. Are you a member of a traumatology committee (local or regional)?
☐ Yes
☐ No

**The questionnaire is over!**

**Please make sure you have answered all the questions.**

**THANK YOU FOR YOUR COLLABORATION**

**If you have any comments or suggestions regarding this questionnaire or this study please write them here:**
